# Implementation of a Hypothesis-Driven Physical Exam Session in a Transition to Clerkship Program

**DOI:** 10.15766/mep_2374-8265.11043

**Published:** 2020-11-24

**Authors:** Julia Kelly, Sandra K. Oza, Richard Feinn, Todd Cassese

**Affiliations:** 1 Resident Physician, Department of Medicine, Tufts Medical Center; 2 Associate Professor, Department of Medicine, Albert Einstein College of Medicine; Co-Director, Introduction to Clinical Medicine and Transition to Clerkship, Albert Einstein College of Medicine; 3 Associate Professor, Department of Medical Sciences, Frank H. Netter MD School of Medicine at Quinnipiac University; 4 Associate Professor, Department of Medicine, Albert Einstein College of Medicine; Assistant Dean for Clinical Sciences Education, Albert Einstein College of Medicine

**Keywords:** Clinical Reasoning, Physical Examination, Hypothesis-Driven Physical Examination, Clinical Skills, Clinical Reasoning/Diagnostic Reasoning, Clinical/Procedural Skills Training

## Abstract

**Introduction:**

The head-to-toe approach to teaching the physical examination (PE) focuses on technique and performing a comprehensive PE whereas core + clusters and hypothesis-driven PE (HDPE) approaches integrate clinical reasoning into performing a focused PE. These approaches can be implemented in a developmental sequence. We report the implementation and evaluation of an HDPE educational session.

**Methods:**

We designed a 3-hour HDPE session as part of a transition to clerkship program. For each of five clinical vignettes, rising third-year students worked in pairs and then in small groups to generate a differential diagnosis and determine relevant PE maneuvers. Students next performed these maneuvers on peers with facilitator observation and feedback. Students completed postsession surveys on their retrospective pre- and postsession knowledge and confidence, as well as their satisfaction with the session. We completed quantitative and qualitative analyses on survey data.

**Results:**

One hundred ninety-two students participated, and 140 (73%) completed the survey. Students were significantly more likely to report feeling confident generating a differential diagnosis and using it to select PE maneuvers for common complaints postsession. Over 80% of respondents felt the session improved critical thinking about patient presentations and would help them in clerkships.

**Discussion:**

Our session increased student confidence in the progression to performing an HDPE just prior to the start of clerkships. The session is feasible and straightforward to implement. It requires a large number of faculty to facilitate, but the breadth of cases used allows inclusion of faculty from all fields.

## Educational Objectives

By the end of this activity, learners will be able to:

1. Generate an appropriate differential diagnosis based on a case vignette.
2. List physical exam maneuvers that correspond to the diagnoses on the differential.
3. Justify the selection of a given physical exam maneuver by detailing the expected findings relevant to the diagnoses on the differential.
4. Practice selected physical exam maneuvers identified in the previous objectives with a partner.

## Introduction

Physical examination (PE) is a vital skill for physicians in all fields to master. A well-executed PE is important for establishing a diagnosis, guiding therapy, determining prognosis, and providing physical contact that can promote the patient-physician relationship.^1^ It accomplishes this in a way that is easily accessible and safe for the patient while simultaneously providing pedagogic value to learners.^1^ Furthermore, the use of PE in some scenarios (e.g., performing a detailed neurologic exam in a patient presenting with low back pain) can reduce the need for diagnostic testing and associated costs.^2^

One approach that some preclerkship physical diagnosis courses take is to teach early clinical learners to perform a series of over a hundred organ system–specific PE maneuvers.^3^ Students in this learning model are then assessed on their ability to reliably perform a comprehensive head-to-toe (HTT) PE, which is typically carried out on a healthy individual.^3^ However, this HTT approach to learning the PE does not integrate the PE maneuvers with the clinical picture at hand. Therefore, some hypothesize that it encourages rote learning of maneuvers without understanding the context for doing them.^4^ When on the wards, students and residents may have difficulty identifying abnormal PE findings and understanding their significance,^5,6^ which may be due to the decontextualization of the PE when taught in this way.^4^

Despite these drawbacks, the HTT approach still plays an important role in teaching PE skills. Early preclinical learners can be taught the HTT approach prior to learning the relevant pathophysiology needed to approach a patient in a hypothesis-driven way.^7^ Additionally, vague clinical presentations may call for a complete exam, and familiarity with the HTT approach may be beneficial in these situations.^7^ Furthermore, there is some thought that the regular repetition of the full PE by medical students allows more opportunity for skill refinement.^7^ Also, it is possible that learning the choreography of how to perform a particular set of PE maneuvers prior to and separate from interpretation of abnormalities may permit early learners to develop motor memory when working with patients in real clinical settings.

Two other methods of teaching PE skills more directly weave clinical reasoning into the performance of the PE. In the core + clusters approach, students learn a set of core maneuvers to be performed on each patient as well as a set of diagnostic clusters consisting of PE maneuvers specific to and limited based on their relevance to a given clinical condition.^8^ This method involves the incorporation of some clinical reasoning as students must select the relevant cluster based on the patient's presentation. However, the need for advanced clinical reasoning is limited because the approach does not require selection of the specific maneuvers (maneuvers are predetermined for each diagnostic cluster). A second approach, called the hypothesis-driven PE (HDPE), requires a more advanced clinical reasoning ability on the part of the learner because, in it, the learner must formulate a list of appropriate PE maneuvers based on a differential diagnosis determined by the presenting history—students identify maneuvers that either support or refute these diagnoses.^9^ A study by Kamel and colleagues showed that a hypothesis-driven approach to a neurologic exam yielded greater sensitivity and a trend toward faster examinations when compared with a traditional screening approach, although it yielded lower specificity, which was not surprising because neurologic findings amongst the patient population were intentionally subtle.^10^

Furthermore, HDPE may be most similar to what practicing physicians are likely to do while carrying out a PE and is more likely to lead to the identification of abnormal PE signs when carried out by physicians.^11^ In a study by Brooks, LeBlanc, and Norman, both physicians and medical students were more likely to be able to identify obvious abnormal physical signs in photographs of patients when primed with a differential diagnosis, thus highlighting the importance of anticipating possible signs rather than just going through the motions of completing a PE.^12^ Similarly, as indicated above, a hypothesis-driven neurologic examination enabled students to more accurately identify subtle neurologic findings than did a screening neurologic examination.^10^

Several educational activities focused on teaching HDPE are described in the literature.^4,9^ However, to our knowledge, the existing literature does not describe purely formative instructional sessions that focus on the diagnostic approach to specific concerns and the subsequent generation of a focused PE tailored to these concerns or sessions aimed at students in the transition from the preclerkship to the clinical curriculum.

Given that the literature demonstrates utility of HTT, core + clusters, and HDPE approaches, the best approach may be a combination of these methods into a longitudinal curriculum that developmentally progresses from HTT to core + clusters to HDPE as learners develop a greater fund of knowledge and progress in their clinical reasoning skills. We aimed to incorporate HDPE into a curriculum beginning with an HTT approach in the first year of medical school and gradually transitioning to core + clusters early in the second year and then HDPE near the end of the second year. This was done by implementing an educational session in a transition to clerkship program occurring between the second and third years. This session engaged students in creating a differential diagnosis based on a simulated patient encounter to guide the selection of PE maneuvers. Students then practiced performing focused HDPEs with direct supervision and provision of feedback from faculty. Our goals for this educational session were to have students practice HDPE maneuver selection and PE skills to increase student confidence in performance of a problem-focused PE imbued with a clinical reasoning scaffolding.

## Methods

### Curricular Context

PE instruction at our institution begins in the first preclerkship year of medical school, when students learn an HTT approach to a core PE through a peer-practice pedagogical model. Students are assessed during this first year on their performance of this core PE separately by faculty and standardized patients in a simulated environment. In the second preclerkship year, students acquire additional PE skills taught using a hybrid core + clusters and HDPE approach in which they are encouraged to employ increasingly complex clinical reasoning skills in determining what goes into a problem-focused PE. Students practice PE skills in a variety of contexts and are assessed on their performance of PE skills by faculty in the clinical environment and by standardized patients during an observed structured clinical encounter (OSCE).

In the 2019–2020 academic year, our institution implemented a new transition to clerkship week for rising third-year students. A needs assessment including student focus groups and a review of existing published and unpublished national curricula informed the curricular template of the transition week and identified priority areas for curriculum development, including PE practice with feedback on technique and opportunities to practice clinical reasoning. We implemented this HDPE session as part of this June 2019 transition week. Session learning objectives were developed in line with previously indicated goals and were geared towards giving students a more formal mental map of how to apply clinical reasoning principles to the performance of a more mindful, streamlined, and deliberate problem-focused PE. These objectives were aligned with broader objectives for the transition to clerkship week to improve student readiness for and confidence in commencing clerkship education. Specific improvement in distinguishing normal from abnormal PE findings was not an objective we had for this learning activity. The five case vignettes covered in this session were developed by faculty educators and chosen to be representative of cases from our institution's core clerkships.

### Implementation

The session was composed of a 1-hour facilitator preparation session and a 3-hour interactive student workshop. During the facilitator training, one of the authors (Sandra K. Oza) reviewed the session goals and objectives, session format, and suggestions for small-group facilitation. Students were assigned to groups of nine to 10 students per facilitator to review five clinical vignettes over the 3-hour session. For each of five clinical vignettes, students did the following:

1. They worked in pairs to brainstorm differential diagnoses, determine the relevant HDPE maneuvers to perform, and establish a rationale for performing each maneuver.
2. They participated in a small-group discussion led by the facilitator focused on:
  • A diagnostic approach to that chief complaint and how that approach could facilitate generation of diagnostic hypotheses,
  • What PE maneuvers would be indicated based on these diagnostic hypotheses, and
  • What specific findings students would be looking for in the performance of those maneuvers and how those findings would be helpful diagnostically.
3. They practiced selected PE maneuvers in pairs, while the facilitator circulated to answer student questions and give feedback on PE performance.

### Session Materials

#### Student worksheet

We designed a worksheet for students to complete and refer to during the session (Appendix A). For each case vignette, the worksheet included a prompt for generating differential diagnoses and a table into which students entered their brainstormed HDPE maneuvers and justified the choice of each maneuver. Each case in the handout was followed by a description of selected PE maneuvers, with both text and visual components demonstrating normal exam technique and/or findings to guide performance of the maneuver during the peer-practice part of the session.

#### Facilitator guide

We created a facilitator guide, which included the same information provided on the student handout as well an introduction to the session, a suggested time line for the session, suggested differential diagnoses and potential approaches to each chief complaint, suggested PE maneuvers with rationale, and a proposed wrap-up section (Appendix B).

### Assessment

Achievement of the goals and objectives for this HDPE session was measured via students’ self-report of knowledge and skills attained and changes in their confidence level for performing these skills. Additionally, facilitators were surveyed on their perceptions of the value of the learning activity and the performance of the students in the session. Faculty educators and local experts in assessment and evaluation developed surveys designed to measure student attainment of the session goals and objectives. Immediately following the HDPE session, students and facilitators completed a survey with Likert rating scale and free-response items evaluating the session (Appendices C and D). Survey items assessed student perceptions of the achievement of session learning objectives (rated on a scale of *Not at All, Slightly, Moderately,* and *Completely*) and relative strengths and weaknesses of the session (rated on a scale of *Strongly Disagree, Disagree, Agree,* and *Strongly Agree*). We also surveyed students about their confidence before and after the session in performing a focused PE on a patient presenting with a variety of chief complaints (rated on a scale of *Not at All, A Little, Moderately,* and *Very*). Free-response questions assessed self-reported perceptions of the utility of the session during future clerkships, ways to improve the session, and areas of difficulty for students. Students also completed a survey (Appendix E) 4 months after the initial assessment to measure their opinion of the effectiveness of this session after spending 4 months on the wards (rated on a scale of *Poor, Satisfactory, Good,* and *Exceptional*) and their current confidence in performing a focused PE on a patient presenting with a variety of chief complaints (rated on a scale of *Not at All, A Little, Moderately,* and *Very*). Our original intention was to have students complete an electronic version of the student worksheet in real time during paired discussion of the differential diagnoses and generation of the HDPE maneuvers with justification, to assess their ability to identify an appropriate differential diagnosis and list of PE maneuvers for the chief complaint as well as appropriately justify the selection of exam maneuvers. However, internet connectivity problems in our educational space precluded our ability to collect such data electronically, and so, students completed paper worksheets. As a result, we could not ensure that the student worksheets represented solely individual (or paired) student data rather than data from the group discussion, and so, we excluded such data from our analyses.

For the analyses of survey data, we collapsed *Not at All* with *A Little* responses as well as *Moderately* with *Very* responses (Table 1), *Moderately* with *Completely* responses (Table 2), and *Strongly Disagree* with *Disagree* responses and *Agree* with *Strongly Agree* responses (Table 3). A priori, we did not expect that a single instructional session would give many students the ability to completely meet all learning objectives nor to all become very confident in their ability to do this complex task of combining clinical reasoning and PE skills, and this guided our combination of ratings. We calculated descriptive statistics of student and facilitator responses, compared retrospective pre/post ratings of student knowledge and confidence using a nonparametric Wilcoxon signed rank test, and determined effect sizes by Cohen's *d* for paired data.

**Table 1.**
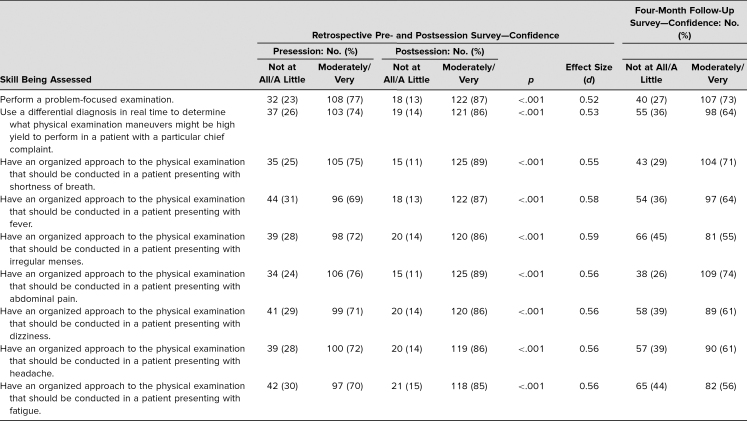
Comparison of Retrospective Pre- and Postsession Confidence in Skill Performance and 4-Month Follow-Up Confidence Levels

**Table 2.**
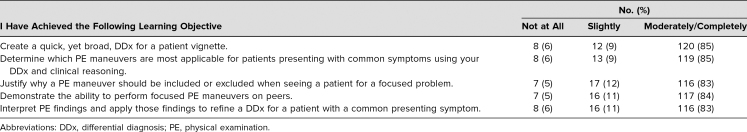
Student Survey Responses Assessing the Achievement of Learning Objectives (*N* = 140)

**Table 3.**
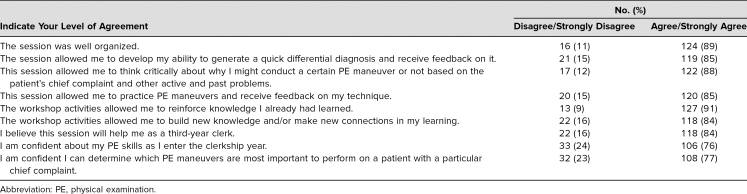
Student Survey Responses Assessing Workshop and PE Skill Confidence (*N* = 140)

We conducted qualitative analyses of student and facilitator comments using a thematic approach. One of the authors (Julia Kelly) reviewed all student comments and generated an initial list of themes. Following this, two other authors (Sandra K. Oza and Todd Cassese) reviewed student comments and the theme list and suggested additional or modified themes. Finally, these three authors met to reconcile differences until consensus was reached on the list of themes.

## Results

### Participants

One hundred ninety-two students (the entire rising third-year class) participated in the HDPE session. One hundred forty students (73%) completed a postsession survey. One hundred fifty students completed all items in the 4-month follow-up survey.

Twenty physicians participated as facilitators (one PGY 2 internal medicine resident, two internal medicine fellows, and 17 faculty members representing a broad range of specialties). Of participating facilitators, 14 (70%) completed a postsession survey.

### Student Evaluation of Session

Students were significantly more likely after than before the workshop to be confident in their ability to perform a problem-focused PE (*p* < .001, *d* = 0.52) and use a differential diagnosis to determine which PE maneuvers to complete (*p* < .001, *d* = 0.53; Table 1). Students were also significantly more likely to express confidence in their ability to have an organized approach to the PE for patients presenting with each of the chief complaints covered in this session, as well as two chief complaints that were not covered in the session (headache and fatigue; Table 1).

More than 80% of students indicated that they had moderately or completely achieved all of the of the expressed learning objectives, with 85% indicating that they learned to make broad differential diagnoses and determine which PE maneuvers were most useful based on the differential diagnosis and clinical reasoning (Table 2). Furthermore, more than three-quarters of students reported that they either agreed or strongly agreed that the session improved their critical thinking about patient presentations and maneuver selection and that this session would help them in clerkships (Table 3).

Thematic analysis of written feedback resulted in the identification of five themes:

1. PE skill learning and reinforcement.
2. Importance of a focused PE.
3. Emphasis on clinical reasoning.
4. Session organization and execution.
5. Session length and timing.

Students indicated that the review of PE skills with feedback and the review of PE maneuver selection were the most beneficial aspects of this session. Multiple students remarked that they learned a new PE skill during this session. Students also indicated that they were able to learn how to approach a PE in a more focused way and were able to consolidate learning from prior years to approach a case as part of this session. Overall, student comments were positive about the organization of the session, including the cases selected, the format of the session, and the student guide, although a number of student comments suggested that the session could be shorter in duration and/or implemented in a longitudinal series. Below, example student comments arranged by theme are presented:

1. PE skill learning and reinforcement:
  • “This session refreshed the physical exam for me, so I foresee using this knowledge in clerkships.”
  • “[One thing I learned during this session was] how to elicit the triceps reflex.”
  • “I learned some new exam maneuvers.”
  • “I re-learned a lot of maneuvers to check for specific abdominal conditions.”
  • “I learned how to measure JVD [jugular venous distention], which was really exciting!”
  • “[A strength of this session was] it reinforced aspects of PE maneuvers that I had forgotten.”
2. Importance of a focused PE:
  • “I learned how important it is to come up with a problem list prior to initiating the physical exam.”
  • “I learned to consider PE maneuvers from a different organ system that may not have been immediately relevant to the primary chief complaint.”
  • “[I can foresee using this in clerkships by] coming up with the differential and thinking about physical exam maneuvers.”
3. Emphasis on clinical reasoning:
  • “[I can foresee using this in clerkships by] beginning to integrate information across organ systems which up until this point have been siloed by the different courses.”
  • “[I can foresee using this in clerkships by] thinking about many different differentials.”
  • “[One thing I learned from this session is the] importance of using algorithms in clinical medicine.”
  • “The strengths of this session were that it engaged us intellectually while forcing us to review a wide-array of high-yield concepts, diagnoses, and maneuvers for the wards.”
  • “I have a much greater appreciation for the physical exam now as a tool to narrow the differential diagnosis and rule out different conditions.”
4. Session organization and execution:
  • “[The session] was very organized and thorough.”
  • “Everything in this session was incredibly relevant for third year. I'm sure I'll use the concepts reviewed in this session each and every day of this year.”
  • “The chief complaints chosen provide a relatively broad overview of what may be encountered on clinical rotations.”
  • “The small groups were extremely useful.”
  • “The level of direction of this session helped it move along well and so it went much better organized than ICM [Introduction to Clinical Medicine] sessions we've done before.”
  • “The session was organized, images were helpful in performing PE maneuvers.”
  • “I liked discussing the scenarios, differentials, and review of PE maneuvers.”
  • “I did appreciate that we had a worksheet, with the exam maneuvers explained.”
  • “[A strength of this session was] the opportunity to talk through the cases with the facilitator.”
5. Session length and timing:
  • “I felt that three hours for a session was a long time and that perhaps the session can be shortened for next time or maybe there can be 5-minute breaks throughout the session.”
  • “I think we need more of these sessions throughout the week to keep reinforcing these concepts. I believe that this session captured much more of what ‘transition to clerkship’ means to me. I was feeling rusty after studying for [USMLE Step 1] for so many weeks, so it was nice to review physical exam stuff and it would be better if we did more of that multiple times during the week so that we felt fully refreshed entering into our rotations.”
  • “[This session could be made better by having] shorter sessions on different days of orientation to go over more differentials and physical exam maneuvers.”
  • “I really enjoyed this session. It was a little long and could probably be split up into two 2-hour sessions with an additional case or two instead of one 3-hour session.”
  • “I think having more of these sessions in a week could be helpful.”
  • “3 hours… a bit much for stuff we spent 2 years on.”
  • “The session should have been a lot shorter… 1.5 hrs max.”
  • “It could have been a little shorter.”
  • “[This session could be made better by having] a shorter session.”
  • “[This session could be made better by having] three cases over 2 hours, the session is too long.”
  • “The session should be made shorter. 3 hours was way too long and everyone stopped focusing around hour 2.”

In the 4-month follow-up survey, 76% of students reported that this session was satisfactory, good, or exceptional. Student confidence was uniformly lower at the 4-month follow-up than both pre- and postsession confidence levels as measured immediately after the session. On all items, a smaller percentage of students reported feeling moderately or very confident in each skill, with the greatest changes seen in having an organized approach to a patient with irregular menses or fatigue (Table 1).

### Facilitator Evaluation of Session

All facilitators indicated that learning objectives of the session were either moderately or completely met. In addition, all facilitators indicated that they found the facilitator training session and facilitator guide to be moderately or extremely valuable and reported that the time allotted for this session was either just right or ample. Facilitators overall felt that group size (nine to 10) was ideal, and all facilitators indicated that they were either very or extremely satisfied with the experience of teaching in this new session. Analysis of facilitator comments revealed that facilitators found the review of PE skills extremely beneficial for students and that a strength of the session was the explicit emphasis on the clinical reasoning clinicians use when selecting PE maneuvers. Facilitator comments echoed student comments on session duration, noting that student attention decreased at the end of the session.

## Discussion

We introduced an HDPE session promoting clinical reasoning by means of discussion of a diagnostic approach to a clinical problem through differential diagnosis generation paired with selection and justification of PE maneuvers. This session effectively increased student confidence in focused PE maneuver selection. The session was well received by students and faculty in terms of its usefulness for preparing students for clinical clerkships and its overall setup, and students continued to perceive a benefit from the session 4 months into the clerkship year. Explicit opportunities for PE practice reinforced PE skills that students learned during their preclinical years just prior to the start of their time on the wards and after an approximately 2-month study period for USMLE Step 1 during which students did not practice clinical skills. The session was inexpensive to implement due to its use of peer PE practice and volunteer physician facilitators.

The term HDPE has been used to describe many different types of educational sessions in different contexts.^4,9^ Our session is unique because of its sequencing in the curriculum and the specific focus of the learning activity. Yudkowsky and colleagues^4^ worked with third-year students, and their session was used more as a summative assessment during clerkships than a formative learning activity. Nishigori and colleagues^9^ crafted an HDPE learning activity focusing on how different PE maneuvers could be used to discriminate between different prespecified diagnoses. The emphasis of their session was on interpreting PE findings and utilizing them to narrow in on a most likely diagnosis. Our session required students to create a broad differential diagnosis for several sample cases prior to selection of relevant PE maneuvers. This allowed for priming with the potential differential diagnoses, which was found by Brooks, LeBlanc, and Norman to increase the likelihood that certain physical findings will be anticipated and therefore noticed.^12^ Additionally, our session was uniquely positioned as part of a transition to clerkship curriculum, which allowed the session both to serve as an opportunity for revisiting and strengthening existing skills and to help prepare students for the clinical skills demands of the clerkship year. Our student evaluation data support the idea that the session achieved this dual role and confirm the utility of the session in preparing students to meet those challenges.

### Limitations

A limitation of this work is that it was developed and implemented at a single institution as part of a weeklong transition to clerkship curriculum, which could limit generalizability to institutions with different curricular structures. Overall, the session is inexpensive to execute, but it does require facilitator availability to lead the small-group discussion and supervise PE practice, as well as adequate classroom/clinical skills facility space, which could be limiting based on the resources available. At our institution, we found that one facilitator per nine to 10 students was adequate for direct observation and feedback; other ratios may be preferred in other settings. The breadth of clinical vignettes lends itself well to the recruitment of facilitators from a broad range of clinical specialties; however, in order to increase facilitators’ comfort with facilitating cases outside their area of specialty, dedicated faculty development time is needed.

### Lessons Learned and Future Directions

We feel it is essential to view the feedback on this HDPE session in the context of the at times negative feedback our institution received from students about the overall transition to clerkship week within which the session was situated. We hypothesize that this was largely due to the sense among students that the transition week had replaced time that would, in previous years, have been allocated as dedicated study time to prepare for USMLE Step 1 and/or vacation. Though not a sentiment shared by all or even most students, it was present in student survey responses such as the following comment: “I feel like it would be much better for my education and personal wellbeing to either have had a spring break, an extra week of dedicated, or an extra week of vacation than to have done this workshop.” Moving forward, we expect that with each year, the transition week and its sessions will be received more positively as students focus on the benefits of the teaching, review, and preparation received during the transition week and are less aware that a particular week of time used to be utilized differently. Due to the impact of the COVID-19 pandemic on our curriculum and our community, we were unable to include this session in the 2020 transition to clerkship (which was run entirely virtually) but hope to be able to repeat it again it in future years.

In our 4-month follow-up results, we noted that over three-quarters of students continued to perceive a benefit from this session during their clerkships, although the percentage of students reporting higher confidence levels in each of the skills assessed in the original survey declined. This decline would be expected several months after a onetime session, particularly in conjunction with the lack of ongoing formal practice of these skills during clinical clerkships. Of note, student confidence levels were lower than both pre- and postsession confidence levels, which might indicate that a factor other than time-related attrition influenced the decline in confidence. One possible factor is the challenging nature of the clinical clerkships, including the adjustment to new roles in a new setting, working with more complex patients, comparison to more advanced providers, or the general deficiency in bedside clinical PE teaching in clerkships.^13^ Future directions include incorporating student feedback to explore a longitudinal series of these sessions that could span the preclerkship clinical skills course and clerkships within the first half of the year. Additionally, we would like to correlate student perception of confidence with performance by incorporating assessment activities of HDPE via simulation (such as with an OSCE) or in the clinical setting (such as a mini-clinical examination done by faculty direct observation) to gain a more objective measure of student ability to select PE maneuvers using an HDPE approach, rather than confidence levels, which can be more subjective. Student data from other formal assessments such as clerkship OSCEs or evaluations could also be linked to student performance in these workshops to compare their abilities in a variety of settings.

There are several ways this session could be improved in the future. Student and faculty comments indicated that session productivity declined at the end of the 3-hour session. In addition to being able to reinforce the content across clerkships and settings, dividing the session into a longitudinal series of shorter sessions over time would also ameliorate the fatigue setting in near the end of a lengthy session that challenges learners from start to finish. The use of multiple shorter sessions could also be more engaging and would allow for spaced repetition of the concepts. Spaced learning has been shown to increase students’ long-term retention of material^14^ and has been utilized for teaching PE to medical students, resulting in significant improvement in knowledge of the PE.^15^ Therefore, it is possible that additional sessions would also help to address the decline in student confidence at the 4-month mark. If this longitudinal approach were incorporated, difficulty of cases or concepts could be increased progressively over the clerkship period, and the amount of guidance provided by facilitators might naturally decrease as students gain competence.

Aspects of this session could be incorporated into bedside teaching on the wards by taking time between history and PE for the faculty and student to discuss the differential diagnosis and possible relevant PE maneuvers. This approach could be incorporated into formative or summative OSCEs. The deconstruction of the thought process behind PE maneuver selection could allow for a better understanding of the student's clinical reasoning process and therefore more constructive feedback on these assessments. An added advantage of utilizing real patients would be that the anticipation and recognition of abnormal physical findings could be woven into the instruction. In our session, students performed PE maneuvers on (generally healthy) peers. While students were primed during the discussion to anticipate abnormal findings (i.e., the justification step of “What are you looking for?”), the use of real patients would enable students to take the next step in recognizing the presence of such findings. Incorporation of this approach amongst our clinical teachers would require faculty interest, engagement, and development, but given the positive response of our faculty preceptors in teaching in this session, there is a clear path towards broader implementation in this manner.

While our session was designed for incorporation into a transition to clerkship curriculum, it could be modified to fit the needs/resources of other education settings, such as preclerkship courses or clinical clerkships, or adapted to incorporate other educational resources. For example, the session could include standardized patients instead of utilizing peer PE practice if the resources are available. It could also be adapted into a group OSCE with the same cases followed by the same discussion of the PE selection based on differential diagnosis as utilized currently.

### Conclusions

Our HDPE session effectively increased student confidence in performing a focused PE. This relatively inexpensive and easily implementable session can be modified to fit the needs of a given school and is an effective way to promote students’ progression from HTT-based PE to HDPE as they enter their clinical clerkships.

## Appendices

Student Worksheet.docxFacilitator Guide.docxPostsession Student Survey.docxPostsession Facilitator Survey.docxFour-Month Follow-Up Student Survey.docx
All appendices are peer reviewed as integral parts of the Original Publication.
