# Supplementary material for: Implementation of a Hypothesis-Driven Physical Exam Session in a Transition to Clerkship Program
Source: MedEdPORTAL. 2020 Nov 24;16:11043. doi: 10.15766/mep_2374-8265.11043 (PMC7703480; doi:10.15766/mep_2374-8265.11043)
Supplement: Supplementary file 1 — Student Worksheet.docxFacilitator Guide.docxPostsession Student Survey.docxPostsession Facilitator Survey.docxFour-Month Follow-Up Student Survey.docx [file mep_2374-8265.11043-s001.zip › A. Student Worksheet.docx]

HDPE

Case #1: Shortness of breath

| **Physical Examination Practice: Please work with a peer or two in your small group to practice the following physical examination maneuvers relevant to this case:**   1. Assess for Jugular Venous Distention 2. Perform the following components of the cardiac exam:    1. Auscultate with bell and diaphragm in 4 auscultatory areas    2. Assess for the Point of Maximal Impulse (PMI) 3. Perform the following components of the pulmonary exam:    1. Auscultate the posterior and anterior chest for breath sounds with patient breathing through open mouth.    2. Practice percussion over the posterior lung fields 4. Assess the extremities for cyanosis, clubbing and edema   ***FACILITATOR NOTES: Suggestions for performance of selected PE maneuvers follows below.***  ***Assess for Jugular Venous Distention:*** To visualize the jugular wave forms, have the patient lie flat with the bed tilted to about 25 degrees from horizontal. Next shine a penlight tangentially across the patient’s neck on the side you are examining. If you are examining the right side of the patient’s neck use your right hand and if you are examining the left side use your left hand. The light will create shadows of the jugular venous pulsations onto the sheet that the patient is lying on.^1^  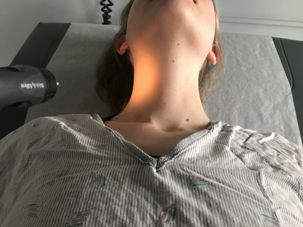  ***Photo: Sandra K. Oza, MD, MA***  ***Auscultate with bell and diaphragm in 4 auscultatory areas:*** Begin by assessing the 4 auscultatory areas while the patient is supine, moving sequentially from the aortic area to the mitral area (illustrated below). Next, have the patient lie on their left side and auscultate the mitral area using the bell of the stethoscope, which will elicit the diastolic rumble of mitral stenosis if present. Next, examine all 4 cardiac areas with the patient upright. Conclude by listening to the right and left second and third intercostal spaces while the patient leans forward and exhales or holds their breath. This will elicit the soft high-pitched decrescendo murmur of aortic regurgitation if present.^2^  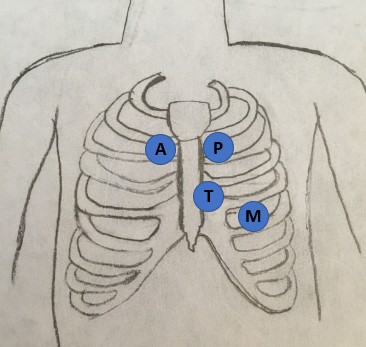  ***Illustration: Julia Kelly, BS***  ***Assess for the Point of Maximal Impulse (PMI):*** The patient should be seated and the examiner should stand to the right of the patient. The examiner’s fingertips should be applied to the patient’s 5^th^ intercostal space in the midclavicular line, adjusting as needed until the PMI is located.^3^  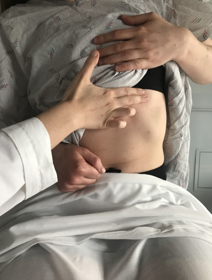  ***Photo: Sandra K. Oza, MD, MA***  ***Assess the extremities for clubbing:*** Ask the patient to place their thumbs together with fingernails touching. Observe the angle formed between the nail base and the finger. If there is no clubbing the angle should be about 160 degrees but when there is clubbing the angle is greater than 180. Additionally, in a nail with clubbing the nail bed becomes spongy and the nail has a more bullous shape.^4^  Finger Clubbing  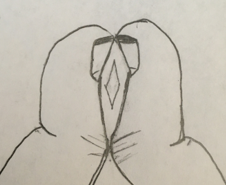 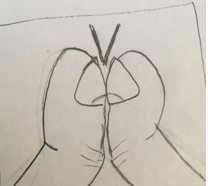  Normal Clubbing  ***Illustrations: Julia Kelly, BS*** |
| --- |

HDPE
Case #2: Abdominal Pain

| **Clinical Vignette:**  43-year-old woman presents with 2 days of abdominal pain. She endorses some mild nausea and has had a single episode of non-bloody, non-bilious emesis. |
| --- |

| **1. Using the information available to you above, please list some potential diagnoses that could lead to this presentation.**  **2. Hypothesis-Driven PE: Using the list of diagnoses you identified above for this patient, which physical examination maneuvers would you plan to perform on this patient? Please fill out the table below justifying why you would be doing that maneuver and what you’d be looking for? You do not need to fill in the entire table, and can add rows if needed.**   \| PE Maneuver \| Justification (what are you looking for)? \| \| --- \| --- \| \|  \|  \| \|  \|  \| \|  \|  \| \|  \|  \| \|  \|  \| \|  \|  \| \|  \|  \| |
| --- | --- | --- | --- | --- | --- | --- | --- | --- | --- | --- | --- | --- | --- | --- | --- | --- |

| **Physical Examination Practice: Please work with a peer in your small group to practice the following physical examination maneuvers relevant to this case:**   1. Perform a HEENT examination – presence of scleral icterus, mucous membrane hydration 2. Perform an abdominal exam, following these steps:    1. Inspection – looking for skin lesions/rashes, surgical scars, abdominal venous pattern    2. Auscultation – presence or absence of bowel sounds    3. Percussion – dullness versus tympany, estimation of liver size    4. Palpation – location of tenderness, assessment of guarding, rebound tenderness    5. Special maneuvers:       1. Assess for the presence of a Murphy’s sign       2. Assess for costovertebral angle tenderness       3. Special techniques for the evaluation of appendicitis:          1. Assess for tenderness at McBurney’s point          2. Rovsing’s sign          3. Psoas sign          4. Obturator sign 3. Discuss the approach to (but do not practice) a pelvic examination   ***NOTES: Suggestions for performance of selected PE maneuvers follows below.***  ***Assess for the presence of a Murphy’s sign:*** Palpate in the right upper quadrant as the patient simultaneously takes a deep continuous inspiration. If pain is elicited and inspiration ceases during this maneuver this is a positive Murphy’s sign which is suggestive of acute cholecystitis. This is because during inspiration the diaphragm pushes down on the liver and gall bladder and palpation of the inflamed gall bladder causes severe pain and inspiratory arrest.^5^  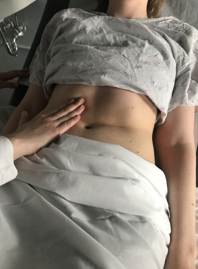 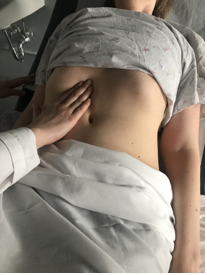  ***Photos: Sandra K. Oza, MD, MA***  ***Assess for costo-vertebral angle tenderness:*** The patient should be seated and the examiner should tap over the costo-vertebral angle with a closed fist. This will cause severe pain in patients with pyelonephritis.^6^  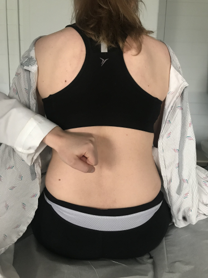  ***Photo: Sandra K. Oza, MD, MA***  ***Assess for tenderness at McBurney’s point:*** McBurney’s point lies 1/3 of the distance between the right ASIS and umbilicus, as illustrated below. Pain at this point is suggestive of acute appendicitis.  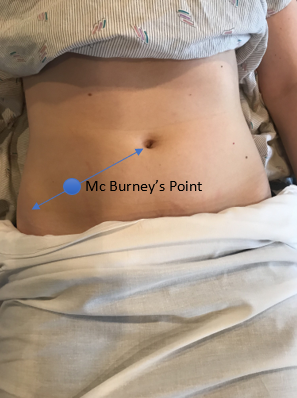  ***Photo: Sandra K. Oza, MD, MA***  ***Rovsing’s sign:*** The patient should be in a supine position and examiner should palpate the left lower quadrant. If this elicits pain in right lower quadrant then this is suggestive of acute appendicitis. ^7^  ***Psoas sign (retroperitoneal retrocecal appendix):***  The patient should be lying on their left side and the examiner should passively extend their right hip. Pain on this maneuver is suggestive of acute appendicitis. ^7^  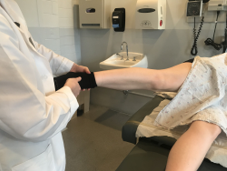 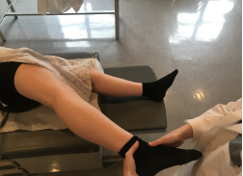  ***Photos: Sandra K. Oza, MD, MA***  ***Obturator sign (pelvic appendix):***  The patient should be in a supine position. The examiner should flex the patient’s right hip and knee, then internally rotate the femur by moving the lower leg laterally while providing resistance to the lateral knee. Pain on this maneuver is suggestive of acute appendicitis. ^7^  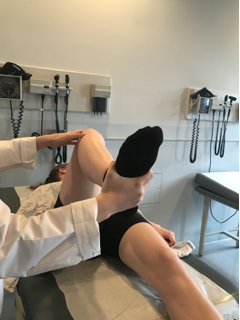 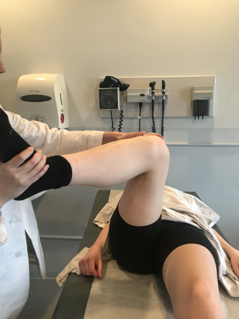  ***Photos: Sandra K. Oza, MD, MA***  ***Discuss the approach to (but do not practice) a pelvic examination:*** Review the steps of the pelvic examination with students that they learned in the ICM-2 course. They were taught the 3 main components of the pelvic exam.   1. Inspection and palpation of the vulva: Assess all structures    1. Mons pubis    2. Labia majora    3. Labia minora    4. Perineum 2. Speculum examination:    1. Technique for insertion       1. Inform patient before beginning, ask patient to bear down       2. Separate labia with index and middle fingers of one hand       3. Choose correct size speculum with other hand       4. Handles are posterior at 45 degrees       5. Insert closed, lubricated speculum through introitus to full length       6. Rotate speculum back 45 degrees until handles in posterior position    2. Visualize and inspect cervix, vagina 3. Bimanual digital examination    1. Labia gently parted with index and middle finger of one hand    2. Lubricated index and middle finger of other hand inserted into vaginal canal    3. Assess vaginal walls, locate cervix    4. Assess cervix, uterus, adnexal structures |
| --- |
|  |

HDPE
Case #3: Child with fever

| **Clinical Vignette**  A 6-year-old girl with cerebral palsy who is non-verbal and wheelchair bound presents with 1 day of fever and fatigue. |
| --- |

| **1. Using the information available to you above, please list some potential diagnoses that could lead to this presentation.** |
| --- |

| **2. Hypothesis-Driven PE: Using the list of diagnoses you identified above for this patient, which physical examination maneuvers would you plan to perform on this patient? Please fill out the table below justifying why you would be doing that maneuver and what you’d be looking for? You do not need to fill in the entire table, and can add rows if needed.**   \| Physical Exam Maneuver \| Justification (what are you looking for?) \| \| --- \| --- \| \|  \|  \| \|  \|  \| \|  \|  \| \|  \|  \| \|  \|  \| \|  \|  \| \|  \|  \| \|  \|  \| \|  \|  \| \|  \|  \| |
| --- | --- | --- | --- | --- | --- | --- | --- | --- | --- | --- | --- | --- | --- | --- | --- | --- | --- | --- | --- | --- | --- | --- |

| **Physical Examination Practice: Please work with a peer in your small group to practice the following physical examination maneuvers relevant to this case:**   1. Examine the outer ear and then the tympanic membrane using an otoscope and speculum. 2. Examine the oropharynx using a tongue depressor. 3. Palpate cervical lymph nodes 4. Perform Kernig and Brudzinki signs 5. Examine the skin for rashes. Discuss with your partner where you might look for skin breakdown (sacral, back of head/ears, heels) Note- students will not check each other’s sacral area.   ***NOTES: Suggestions for performance of selected PE maneuvers follows below.***  ***Examine the tympanic membrane:*** Students may hold the otoscope either in the dominant hand, or in the same hand as the ear they are about to examine. With the other hand pull the pinna up, out, and back. With the otoscope hand brace the 5th digit on the face of the patient and insert the otoscope while visualizing the canal.^8^  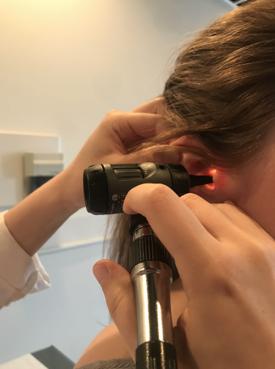  ***Photo: Sandra K. Oza, MD, MA***  ***Examine the oropharynx using a tongue depressor:*** Ask the patient to open their mouth widely while sticking out their tongue. Using a tongue depressor, the examiner should press down on the middle third of the tongue, scooping it towards the front teeth. Putting the tongue depressor too far back may stimulate a gag reflex. Be cautious of avoiding compressing the patient’s lower lip with the anterior portion of the tongue depressor. Use a light source to examine the patient’s oropharynx.^9^  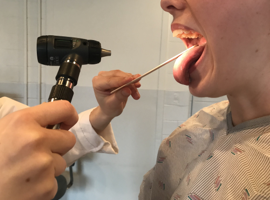  ***Photo: Sandra K. Oza, MD, MA***  ***Palpate cervical lymph nodes:*** The examiner should use the pads of their fingers in a circular motion to palpate the cervical lymph nodes. First palpate the occipital area. Next move to the posterior auricular area, posterior triangle, and along the sternocleidomastoid muscle (first superficially then hooking around the sternocleidomastoid). Finally palpate the anterior triangle region, the submaxillary chain under the jaw, the submental area under the chin, and the anterior auricular area in front of the ear.^10^  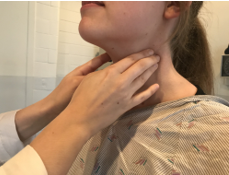 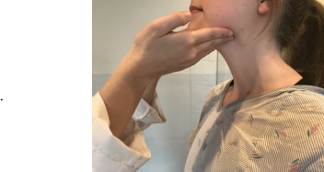  ***Photos: Sandra K. Oza, MD, MA***  ***Perform Kernig’s and Brudzinski’s signs***: For Kernig’s sign the patient should be in a supine position. The examiner should flex the patient’s hip to 90 degrees and attempt to straighten this leg at the knee. Inability to straighten the leg is suggestive of meningitis. For Brudzinski sign the patient should also be supine. The patient’s neck should be passively flexed. A positive sign would occur if neck flexion causes the patients hips and knees to flex. This is suggestive of meningitis.^11^  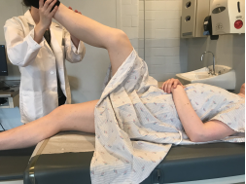 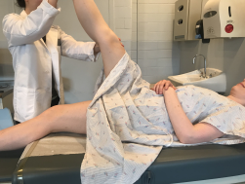  **Checking for Kernig’s Sign**  ***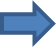*** 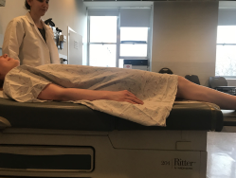 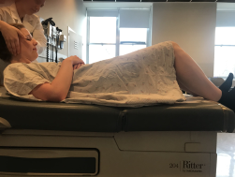  **Positive Brudzinski Sign**  ***Photos: Sandra K. Oza, MD, MA*** |
| --- |

HDPE
Case #4: Irregular Menses

| **Clinical Vignette:**  A 29-year-old woman with no past medical history presents with several months of irregular menses. Menses started at age 12 and were regular, occurring monthly, and lasting 4 days. 6 months ago the patient experienced heavy menstrual bleeding lasting 7 days. She subsequently did not have menses for 8 weeks, and again had a heavy menses for 7 days. She has only had menses one other time since then and it was light and only lasted 2 days. |
| --- |

| **1. Using the information available to you above, please list some potential diagnoses that could lead to this presentation.** |
| --- |

| **2. Hypothesis-Driven PE: Using the list of diagnoses you identified above for this patient, which physical examination maneuvers would you plan to perform on this patient? Please fill out the table below justifying why you would be doing that maneuver and what you’d be looking for? You do not need to fill in the entire table, and can add rows if needed.**     \| Physical Exam Maneuver \| Justification (what are you looking for?) \| \| --- \| --- \| \|  \|  \| \|  \|  \| \|  \|  \| \|  \|  \| \|  \|  \| \|  \|  \| \|  \|  \| \|  \|  \| \|  \|  \| |
| --- | --- | --- | --- | --- | --- | --- | --- | --- | --- | --- | --- | --- | --- | --- | --- | --- | --- | --- | --- | --- |

| **Physical Examination Practice: Please work with a peer in your small group to practice the following physical examination maneuvers relevant to this case:**   1. Assess visual fields by confrontation 2. Perform an examination of the thyroid 3. Assess deep tendon reflexes   ***NOTES: Suggestions for performance of selected PE maneuvers follows below.***  ***Assess visual fields by confrontation***: Begin by sitting at eye level across from the patient, approximately 3 feet away. Both patient and examiner should focus on the other’s nose. The patient should cover one eye and the examiner should close their opposite eye. Next, the examiner will hold up both hands, slightly closer to themselves than the patient. The examiner should show one or two fingers in both hands and ask the patient how many fingers they see. The examiner should be able to see their own fingers during this process. This should be done for upper and lower visual fields for both eyes.^12^  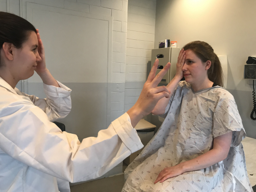 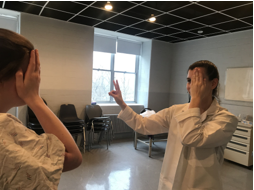  ***Photos: Sandra K. Oza, MD, MA***  ***Perform an examination of the thyroid:***  Anterior Approach: The examiner and patient should sit face to face and the patient should slightly flex their neck towards the side being examined. If examining the right side the examiner should move the larynx to the patient’s left with their left hand. The patient should be asked to swallow while the examiner uses their right hand to palpate for the thyroid on left side of the patients neck between the midline and the left sternocleidomastoid muscle below the thyroid cartilage. This should then be repeated on the opposing side.  Posterior Approach: The examiner should stand to the side of the patient, remaining in their peripheral vision. After describing the exam to the patient, the examiner should place their hands around the patient's neck while slightly extended. If examining the right side of the thyroid the examiner should push the trachea to the right with their left hand. The patient should be asked to swallow while the examiners right hand palpates the area between the midline and the right sternocleidomastoid muscle below the thyroid cartilage. This should then be repeated on the opposing side.^13^  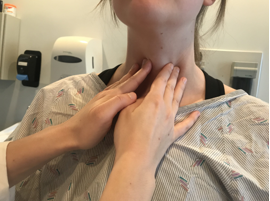 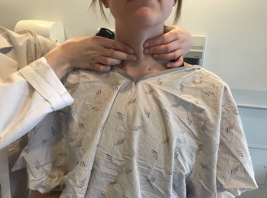  Anterior Approach Posterior Approach  ***Photos: Sandra K. Oza, MD, MA*** |
| --- |

HDPE

Case #5: Dizziness

| **Clinical vignette**:  78-year-old man with past medical history of hypertension, diabetes mellitus type 2, hyperlipidemia, chronic kidney disease stage 3, coronary artery disease s/p MI 3 years ago, BPH presents to urgent care with complaints of one week of dizziness.  Medications:   - Aspirin - Atorvastatin - Hydrochlorothiazide - Insulin glargine - Insulin lispro - Losartan - Metoprolol - Tamsulosin |
| --- |

| **1. Using the information available to you above, please list some potential diagnoses that could lead to this presentation.** |
| --- |

| **2. Hypothesis-Driven PE: Using the list of diagnoses you identified above for this patient, which physical examination maneuvers would you plan to perform on this patient? Please fill out the table below justifying why you would be doing that maneuver and what you’d be looking for? You do not need to fill in the entire table, and can add rows if needed.**   \| PE Maneuver \| Justification (what are you looking for)? \| \| --- \| --- \| \|  \|  \| \|  \|  \| \|  \|  \| \|  \|  \| \|  \|  \| \|  \|  \| \|  \|  \| \|  \|  \| |
| --- | --- | --- | --- | --- | --- | --- | --- | --- | --- | --- | --- | --- | --- | --- | --- | --- | --- | --- |

| **Physical Examination Practice: Please work with a peer in your small group to practice the following physical examination maneuvers relevant to this case:**   1. Measure blood pressure and heart rate; discuss the approach to measuring orthostatic vital signs. 2. Perform the following components of the neurologic examination:    1. Assess cranial nerves    2. Perform a sensory examination of the upper and lower extremities    3. Perform a motor examination of the upper and lower extremities    4. Perform a Romberg maneuver    5. Assess cerebellar function with finger-to-nose and heel-to-shin testing 3. Perform a Dix-Hallpike maneuver   ***NOTES: Suggestions for performance of selected PE maneuvers follows below.***  ***Measure blood pressure and heart rate; discuss the approach to measuring orthostatic vital signs.*** Have the patient lie down for at least 5 minutes and then measure the baseline blood pressure and pulse. Then have the patient stand up and repeat these measurements immediately.  *Orthostatic hypotension* is defined as a drop in systolic blood pressure of 20 mm Hg or more or a drop in diastolic blood pressure of 10 mm Hg or more when a patient stands. The patient also experiences symptoms such as dizziness, lightheadedness, and syncope. There is usually an increase in heart rate.^14^  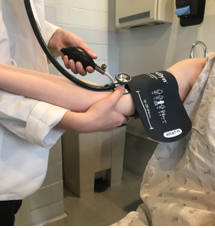  ***Photo: Sandra K. Oza, MD, MA***  ***Perform a Romberg maneuver:*** Have the patient stand with feet together. Stand close to the patient during this maneuver. Ask them to close their eyes. The test is positive if the patient must move their feet to balance and suggests a problem with the dorsal columns. ^15^  ***Assess coordination with finger-to-nose and heel-to-shin testing***:  Finger to Nose: Stand in front of the patient and ask them to touch their nose and then the examiner’s finger, repeating as you move your finger to different points. ^15^  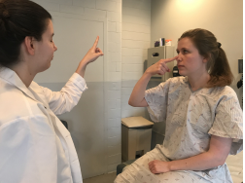 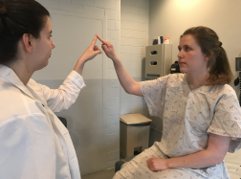  ***Photos: Sandra K. Oza, MD, MA***  Heel to shin: Ask the patient to lie supine and slide the heel of one foot down the opposite leg from knee to ankle. A normal result would be smooth motion of the heel along the shin without removing it from the shin. ^15^  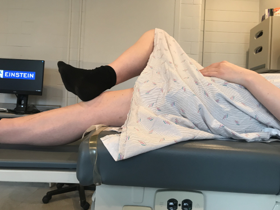 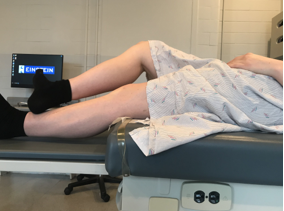  ***Photos: Sandra K. Oza, MD, MA***  ***Perform a Dix-Hallpike maneuver:***  Setup: Begin with the patient seated at the edge of the examining table such that when they lie backwards their head will be slightly off the table behind them. Turn the patients head to 45 degrees from their sagittal plane and to the side being tested. Inform the patient that this may reproduce sensations of dizziness or nausea. Advise the patient to keep their eyes open throughout the maneuver.  Maneuver: Quickly move the patient into a supine position with their head hanging approximately 20 degrees off the table and neck extended slightly so that the chin is up, but maintain their head at the original 45 degree angle to their sagittal plane. Observe the patient in this position for at least 30 seconds, with eyes open. Watch eyes for the direction, duration, and latency of nystagmus. Note if there is any reproduction of symptoms.  Repeat the maneuver with the head turned toward the other side.^16^    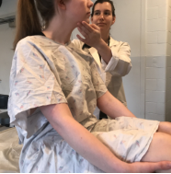 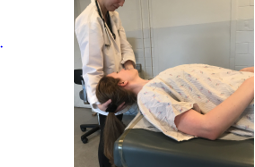 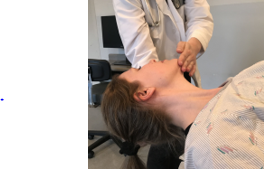  ***Photos: Sandra K. Oza, MD, MA*** |
| --- |

References:

1. Swartz MH. The Heart. In: Swartz MH, ed. *Textbook of physical diagnosis: history and examination*. 7^th^ Ed. Philadelphia, PA: Elsevier Saunders; 2014:370-72
2. Swartz MH. The Heart. In: Swartz MH, ed. *Textbook of physical diagnosis: history and examination*. 7^th^ Ed. Philadelphia, PA: Elsevier Saunders; 2014:376-77
3. Swartz MH. The Heart. In: Swartz MH, ed. *Textbook of physical diagnosis: history and examination*. 7^th^ Ed. Philadelphia, PA: Elsevier Saunders; 2014:373-374
4. Swartz MH. The Skin. In: Swartz MH, ed. *Textbook of physical diagnosis: history and examination*. 7^th^ Ed. Philadelphia, PA: Elsevier Saunders; 2014:90
5. Swartz MH. The Abdomen. In: Swartz MH, ed. *Textbook of physical diagnosis: history and examination*. 7^th^ Ed. Philadelphia, PA: Elsevier Saunders; 2014:452-54
6. Swartz MH. The Abdomen. In: Swartz MH, ed. *Textbook of physical diagnosis: history and examination*. 7^th^ Ed. Philadelphia, PA: Elsevier Saunders; 2014:456-57
7. Hardin DM. Acute Appendicitis: Review and Update. *Am Fam Physician*. 1999 Nov 1;60(7):2027-2034.
8. Swartz MH. The Ear and Nose. In: Swartz MH, ed. *Textbook of physical diagnosis: history and examination*. 7^th^ Ed. Philadelphia, PA: Elsevier Saunders; 2014:266
9. Swartz MH. The Oral Cavity and Pharynx. In: Swartz MH, ed. *Textbook of physical diagnosis: history and examination*. 7^th^ Ed. Philadelphia, PA: Elsevier Saunders; 2014:302
10. Swartz MH. The Head and Neck. In: Swartz MH, ed. *Textbook of physical diagnosis: history and examination*. 7^th^ Ed. Philadelphia, PA: Elsevier Saunders; 2014:149’
11. Bickley LS, Szilagyi PG, Hoffman RM. The Nervous System. In: Bickley LS, Szilagyi PG, Hoffman RM, ed. *Bates’ guide to physical examination and history taking*. 12th Ed. Philadelphia, PA: Wolters Kluwer; 2017:765
12. Swartz MH. The Eye. In: Swartz MH, ed. *Textbook of physical diagnosis: history and examination*. 7^th^ Ed. Philadelphia, PA: Elsevier Saunders; 2014:174
13. Swartz MH. The Skin. In: Swartz MH, ed. *Textbook of physical diagnosis: history and examination*. 7^th^ Ed. Philadelphia, PA: Elsevier Saunders; 2014:153-4
14. Swartz MH. The Heart. In: Swartz MH, ed. *Textbook of Physical Diagnosis: History and Examination*. 7^th^ Ed. Philadelphia, PA: Elsevier Saunders; 2014:366-7
15. Swartz MH. The Nervous System. In: Swartz MH, ed. *Textbook of physical diagnosis: history and examination*. 7^th^ Ed. Philadelphia, PA: Elsevier Saunders; 2014:628-9
16. Munchi H, Sirmans SM, James E. Dizziness: Approach to Evaluation and Management. *Am Fam Physician*. 2017 Feb 1;95(3):154-162.

Notes:
