# Supplementary material for: Implementation of a Hypothesis-Driven Physical Exam Session in a Transition to Clerkship Program
Source: MedEdPORTAL. 2020 Nov 24;16:11043. doi: 10.15766/mep_2374-8265.11043 (PMC7703480; doi:10.15766/mep_2374-8265.11043)
Supplement: Supplementary file 1 — Student Worksheet.docxFacilitator Guide.docxPostsession Student Survey.docxPostsession Facilitator Survey.docxFour-Month Follow-Up Student Survey.docx [file mep_2374-8265.11043-s001.zip › B. Facilitator Guide.docx]

**Transition to Clerkship**

**Hypothesis Driven Physical Examination**

FACILITATOR GUIDE

Dear Faculty:

You will be assigned to a group of approximately 9-10 students. In these small groups you will utilize brief clinical scenario prompts to stimulate student discussion and generation of a differential diagnosis and list of physical examination maneuvers to further evaluate that chief complaint. The learning objectives of the session are for students to:

1. Generate an appropriate differential diagnosis based on a case vignette
2. List physical exam maneuvers which correspond to the diagnoses on the differential
3. Justify the selection of a given physical exam maneuver by detailing the expected findings relevant to the diagnoses on the differential
4. Practice selected physical exam maneuvers identified

For each case, we suggest you ask one student to read the brief vignette. Students then break up into two groups of 4-5 students, and for about 10 minutes will discuss and complete response to prompts 1&2. After 10 minutes, convene a large group discussion of student responses to prompts 1&2 for about 10 minutes. Then, move on to physical exam practice for 10 minutes. Ask students to break up into pairs (or, at most triads) to practice the suggested physical examination maneuvers for this case. While students are practicing, your role is to circulate around to answer questions as well as observe and provide feedback on physical examination technique.

We hope that you find the session to be a fun and engaging learning experience for the students. We are so grateful for your participation and enthusiastic teaching!

FACILITATOR GUIDE TABLE OF CONTENTS

Case #1: Shortness of breath………………………………………………….…………..…………………..3

Case #2: Abdominal pain…………………………………………………………….……………..……………8

Case #3: Child with fever…………………………………………………….…….……….…………….…...17

Case #4: Irregular menses………………………………………………………………………………………23

Case #5: Dizziness……………………………………………………………………………………………….….28

Wrap-up……………………………………………………………………………………………………….….…….34

References……………………………………………………………………………………………………………..35

HDPE

Case #1: Shortness of breath

***FACILITATOR NOTES: For this first case, students will have had an orientation to today’s HDPE session. The content in red was reviewed at the large group session. After doing brief introductions and ice-breakers with your student group, consider spending a few minutes answering any questions that students may have had about this case from the orientation session. At 2:20pm, please ask students to break up into pairs (or, at most triads) to practice the suggested PE maneuvers for this case. While students are practicing, your role is to circulate around to answer questions as well as observe and provide feedback on physical examination technique.***

| **Clinical Vignette**  A 72-year-old man with a past medical history of hypertension, hyperlipidemia, tobacco use and gout presents with a few days of worsening dyspnea on exertion. |
| --- |

| 1. **Using the information available to you above, please list some potential diagnoses that could lead to the presentation above.**   *CHF (systolic or diastolic), cardiac ischemia/angina, pulmonary embolism, anemia, COPD/asthma, pulmonary fibrosis/interstitial lung disease, arrhythmia (others are possible, but the list essentially works out to disease of the pulmonary, cardiac, or hematologic systems)* |
| --- |

***Discuss with students possible frameworks for generating a differential diagnosis?***

*One way I would consider facilitating this is by reminding them of the equation for content of arterial oxygen and delivery of oxygen: DO2 = CO X CaO2 (1.34 X hgb X saturation + 0.003 X pO2). In other words, for tissues not to be hypoxic, they need appropriate cardiac output, hgb and O2 saturation. I can then ask them to think about what lowers CO (CHF, arrhythmia, MI), what lowers hgb (causes of anemia), what lowers sat? (pulmonary causes of hypoxemia)*

| 1. **In thinking through the list of diagnoses you are considering for this patient, which physical examination maneuvers would you plan to perform on this patient? Please fill out a bulleted list justifying why you would be doing that maneuver and what you’d be looking for?**   **Facilitator Note: this information is included just so you know what activity the students completed. When students complete this activity, they will have empty spaces in the table below to fill in.**   \| Physical Exam Maneuver \| Justification (what are you looking for?) \| \| --- \| --- \| \| *Vital Signs* \| *BP – low BP might be a cause of shortness of breath (low CO). High BP might indicate heart failure or increased adrenergic surge.*  *HR- tachycardia could be indicative of PE or adrenergic surge related to cardiac disease or arrhythmia. Bradycardia could also lead to dyspnea.*  *O2 Sat- a low sat would go along with hypoxemia, from pulmonary parenchymal or vascular disease or edema. Normal O2 sats may point more towards anemia.* \| \| *General Exam* \| *Respiratory pattern/presence of distress:*  *How severe/acute is the presentation?* \| \| *External Eye* \| *Conjunctival pallor, scleral icterus (hemolysis) – both related to anemia.* \| \| *Neck* \| *JVP – CHF exacerbation*  *Thyroid exam (airway compression v. CHF)*  *Lymph node exam- perhaps important, perhaps not – is malignancy a possibility?* \| \| *Cardiac* \| *Auscultation, PMI, Hepatojugular reflux – murmurs associated with heart failure or as cause, abnormal rhythm. PMI for cardiomegaly.*  *HJR to assess for increased right sided filling pressures.* \| \| *Respiratory* \| *Auscultation, percussion only if abnormality present. – pulmonary parenchymal disease or pleural disease (effusion, pneumothorax).* \| \| *GI/Abdomen* \| *Pulsatile liver/hepatomegaly – congestive heart failure.*  *Rectal Exam- anemia from overt or occult GI Bleeding.* \| \| *Extremities* \| *Clubbing, cyanosis or edema – clubbing as consequence of chronic hypoxemia. Cyanosis for hypoxemia, edema- heart failure.* \| \| *Skin exam* \| *Perioral cyanosis, digital cyanosis, skin pallor, jaundice. – all related to anemia or hypoxemia.* \| |
| --- | --- | --- | --- | --- | --- | --- | --- | --- | --- | --- | --- | --- | --- | --- | --- | --- | --- | --- | --- | --- |

***Facilitator Note: Everything above was covered in large group. The small group session begins with the activity below!***

| **Physical Examination Practice: Please work with a peer or two in your small group to practice the following physical examination maneuvers relevant to this case:**   1. Assess for Jugular Venous Distention 2. Perform the following components of the cardiac exam:    1. Auscultate with bell and diaphragm in 4 auscultatory areas    2. Assess for the Point of Maximal Impulse (PMI) 3. Perform the following components of the pulmonary exam:    1. Auscultate the posterior and anterior chest for breath sounds with patient breathing through open mouth.    2. Practice percussion over the posterior lung fields 4. Assess the extremities for cyanosis, clubbing and edema   ***FACILITATOR NOTES: Suggestions for performance of selected PE maneuvers follows below.***  ***Assess for Jugular Venous Distention:*** To visualize the jugular wave forms, have the patient lie flat with the bed tilted to about 25 degrees from horizontal. Next shine a penlight tangentially across the patient’s neck on the side you are examining. If you are examining the right side of the patient’s neck use your right hand and if you are examining the left side use your left hand. The light will create shadows of the jugular venous pulsations onto the sheet that the patient is lying on.^1^  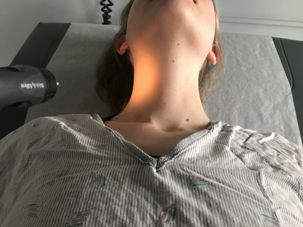  ***Photo: Sandra K. Oza, MD, MA***  ***Auscultate with bell and diaphragm in 4 auscultatory areas:*** Begin by assessing the 4 auscultatory areas while the patient is supine, moving sequentially from the aortic area to the mitral area (illustrated below). Next, have the patient lie on their left side and auscultate the mitral area using the bell of the stethoscope, which will elicit the diastolic rumble of mitral stenosis if present. Next, examine all 4 cardiac areas with the patient upright. Conclude by listening to the right and left second and third intercostal spaces while the patient leans forward and exhales or holds their breath. This will elicit the soft high-pitched decrescendo murmur of aortic regurgitation if present.^2^  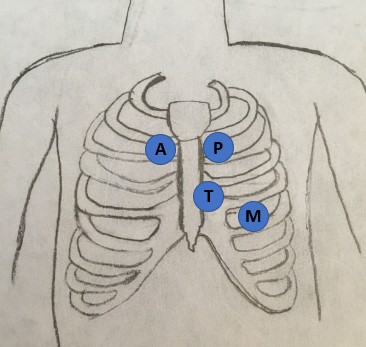  ***Illustration: Julia Kelly, BS***  ***Assess for the Point of Maximal Impulse (PMI):*** The patient should be seated and the examiner should stand to the right of the patient. The examiner’s fingertips should be applied to the patient’s 5^th^ intercostal space in the midclavicular line, adjusting as needed until the PMI is located.^3^  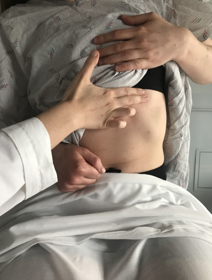  ***Photo: Sandra K. Oza, MD, MA***  ***Assess the extremities for clubbing:*** Ask the patient to place their thumbs together with fingernails touching. Observe the angle formed between the nail base and the finger. If there is no clubbing the angle should be about 160 degrees but when there is clubbing the angle is greater than 180. Additionally, in a nail with clubbing the nail bed becomes spongy and the nail has a more bullous shape.^4^  Finger Clubbing  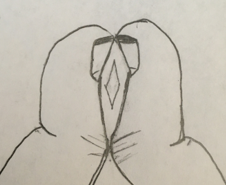 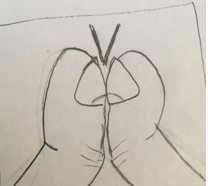  Normal Clubbing  ***Illustrations: Julia Kelly, BS*** |
| --- |

HDPE
Case #2: Abdominal Pain

***FACILITATOR NOTES: Ask one student to volunteer to read the clinical vignette for the group. Once the vignette has been read, ask the students to break up into two groups of 4-5 students to complete the responses to prompts 1-2 on the worksheet. After 10 minutes, convene a full group discussion of student responses to prompts 1-2 for about 10 minutes. Then, move on to physical exam practice for 10 minutes. Ask students to break up into pairs (or, at most triads) to practice the suggested physical examination maneuvers for this case. While students are practicing, your role is to circulate around to answer questions as well as observe and provide feedback on physical examination technique.***

| **Clinical Vignette:**  43-year-old woman presents with 2 days of abdominal pain. She endorses some mild nausea and has had a single episode of non-bloody, non-bilious emesis. |
| --- |

| **1. Using the information available to you above, please list some potential diagnoses that could lead to this presentation.**  ***FACILITATOR NOTES:***  ***During the large group discussion, ask each student to provide one possible diagnosis. Do 1-2 rounds time permitting. Following the large group discussion of a differential diagnosis, discuss with students possible frameworks for generating a differential diagnosis?***  *There are several approaches one can take in the evaluation of “abdominal pain.” Attending to the acuity (vs chronicity) and location (i.e. quadrant) of the pain can help in the generation of an initial differential diagnosis. The clinical vignette is intentionally vague regarding details about the abdominal pain, and we hope that this encourages students to think very broadly/generally about abdominal pain. Of course, additional history would be helpful in determining the most likely of the diagnoses in this table.*  *In ICM-1, students learn the physical exam concurrently with their anatomy course. Consequently, this is the framework and context that our students have in their initial education in the physical exam. We encourage you to take this opportunity to remind students of the importance, when approaching a patient with an abdominal complaint, to consider the underlying anatomy. Localized pain or other symptoms should prompt students to consider what “lies beneath” and may be involved with the symptoms. Discuss also how the underlying biological sex (and thus underlying reproductive organs) is important to consider. How might they have approached this case in someone who is biologically male? This case also serves as a good reminder that, in caring for patients whose gender identity may differ from their biological sex, obtaining a history of prior medical or surgical treatment is important in the approach to abdominal pain in a transgender person.*   \| *RUQ:*   - *Acute hepatitis* - *Acute cholecystitis* - *Acute cholangitis* - *RLL pneumonia* - *R. pyelonephritis* - *Zoster* \| *Epigastric:*   - *Peptic ulcer disease* - *Myocardial infarction* - *Pericarditis* - *Esophageal spasm* - *Gastroesophageal reflux* - *Gastritis* \| *LUQ:*   - *Left pyelonephritis* - *Splenic infarct* - *Peptic ulcer disease* - *Zoster* \| \| --- \| --- \| --- \| \|  \| *Generalized:*   - *Acute pancreatitis* - *Small bowel obstruction* - *Peritonitis* - *Ruptured AAA* - *Acute mesenteric ischemia* - *Acute porphyria* \|  \| \| *RLQ:*   - *Acute appendicitis* - *Pelvic inflammatory disease* - *Ovarian torsion* - *Tubo-ovarian abscess* - *Zoster* - *Nephrolithiasis* - *Ectopic pregnancy* \| *Suprapubic:*   - *Urinary tract infection* \| *LLQ:*   - *Acute diverticulitis* - *Pelvic inflammatory disease* - *Ovarian torsion* - *Tubo-ovarian abscess* - *Ectopic pregnancy* - *Nephrolithiasis* - *Zoster* \| |
| --- | --- | --- | --- | --- | --- | --- | --- | --- | --- |

| **2. Hypothesis-Driven PE: Using the list of diagnoses you identified above for this patient, which physical examination maneuvers would you plan to perform on this patient? Please fill out the table below justifying why you would be doing that maneuver and what you’d be looking for? You do not need to fill in the entire table, and can add rows if needed.**  ***FACILITATOR NOTES: During the large group discussion, ask each student to provide one PE maneuver with justification. Do 1-2 rounds time permitting. A suggested approach to the physical examination for the above case, along with a justification for each of the PE maneuvers, follows below. Encourage students to be as specific as possible with the justification for each of the PE maneuvers they recommend in the evaluation of this patient.***   \| PE Maneuver \| Justification (what are you looking for)? \| \| --- \| --- \| \| *General inspection* \| *With some acute abdominal processes (i.e. ruptured AAA, acute pancreatitis) patients may present with altered levels of consciousness and/or may be in acute distress*  *Intra-abdominal infections may also present with confusion/delirium, which can be identifying by assessing orientation* \| \| *Vital signs* \| *BP – hypotension may be associated with infections, ruptured AAA; hypertension may be associated with MI*  *HR – tachycardia may be associated with infection, peritonitis, MI, pericarditis*  *Temp – fever may be present with infection*  *RR – may be increased in pneumonia, fever, sepsis* \| \| *HEENT exam* \| *Scleral icterus – acute hepatitis, acute cholangitis*  *Oropharynx – dry mucous membranes, sublingual icterus* \| \| *Cardiac exam* \| *Auscultation for:*   - *Rhythm: arrhythmias (i.e. Atrial Fibrillation) as risk factor for acute mesenteric ischemia* - *Extra heart sounds: rubs (pericarditis), new murmurs (MI)* \| \| *Pulmonary exam* \| *Percussion for dullness (suggestive of a PNA)*  *Palpation: for tactile fremitus (increased in PNA, decreased in pleural effusion)*  *Auscultation: breath sounds, assess for egophany* \| \| *Abdominal exam* \| *Inspect: distention, scars, rash/skin lesions (Zoster)*  *Auscultate: bowel sounds (SBO), abdominal bruit (ruptured AAA)*  *Percussion: dullness vs tympany*  *Palpation: location of tenderness, assess for voluntary/involuntary guarding, rebound tenderness*  *Special maneuvers:*  *Murphy’s – assessing for cholecystitis*  *Costovertebral angle tenderness – assessing for pyelonephritis*  *Appendicitis:*  *Tenderness at McBurney’s point*  *Rovsing’s sign*  *Psoas sign*  *Obturator sign* \| \| *Pelvic exam* \| *Cervical inspection: assess for cervical erythema, friability, discharge (PID)*  *Cervical motion: assess for tenderness (PID)*  *Adnexal palpation: assess for tenderness, mass (PID, ectopic pregnancy, tubo-ovarian abscess)* \| |
| --- | --- | --- | --- | --- | --- | --- | --- | --- | --- | --- | --- | --- | --- | --- | --- | --- |

| **Physical Examination Practice: Please work with a peer in your small group to practice the following physical examination maneuvers relevant to this case:**   1. Perform a HEENT examination – presence of scleral icterus, mucous membrane hydration 2. Perform an abdominal exam, following these steps:    1. Inspection – looking for skin lesions/rashes, surgical scars, abdominal venous pattern    2. Auscultation – presence or absence of bowel sounds    3. Percussion – dullness versus tympany, estimation of liver size    4. Palpation – location of tenderness, assessment of guarding, rebound tenderness    5. Special maneuvers:       1. Assess for the presence of a Murphy’s sign       2. Assess for costovertebral angle tenderness       3. Special techniques for the evaluation of appendicitis:          1. Assess for tenderness at McBurney’s point          2. Rovsing’s sign          3. Psoas sign          4. Obturator sign 3. Discuss the approach to (but do not practice) a pelvic examination   ***FACILITATOR NOTES: Suggestions for performance of selected PE maneuvers follows below.***  ***Assess for the presence of a Murphy’s sign:*** Palpate in the right upper quadrant as the patient simultaneously takes a deep continuous inspiration. If pain is elicited and inspiration ceases during this maneuver this is a positive Murphy’s sign which is suggestive of acute cholecystitis. This is because during inspiration the diaphragm pushes down on the liver and gall bladder and palpation of the inflamed gall bladder causes severe pain and inspiratory arrest.^5^  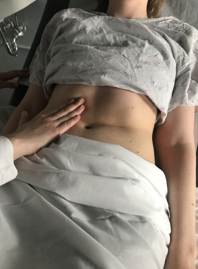 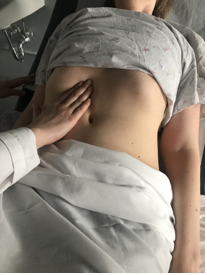  ***Photos: Sandra K. Oza, MD, MA***  ***Assess for costo-vertebral angle tenderness:*** The patient should be seated and the examiner should tap over the costo-vertebral angle with a closed fist. This will cause severe pain in patients with pyelonephritis.^6^  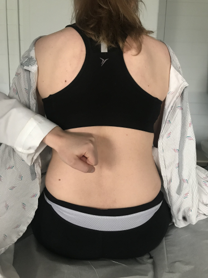  ***Photo: Sandra K. Oza, MD, MA***  ***Assess for tenderness at McBurney’s point:*** McBurney’s point lies 1/3 of the distance between the right ASIS and umbilicus, as illustrated below. Pain at this point is suggestive of acute appendicitis.  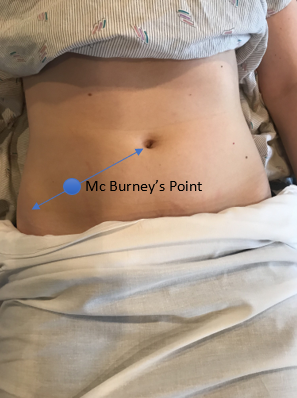  ***Photo: Sandra K. Oza, MD, MA***  ***Rovsing’s sign:*** The patient should be in a supine position and examiner should palpate the left lower quadrant. If this elicits pain in right lower quadrant then this is suggestive of acute appendicitis. ^7^  ***Psoas sign (retroperitoneal retrocecal appendix):***  The patient should be lying on their left side and the examiner should passively extend their right hip. Pain on this maneuver is suggestive of acute appendicitis. ^7^  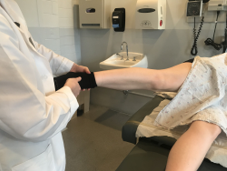 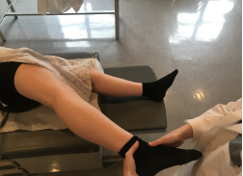  ***Photos: Sandra K. Oza, MD, MA***  ***Obturator sign (pelvic appendix):***  The patient should be in a supine position. The examiner should flex the patient’s right hip and knee, then internally rotate the femur by moving the lower leg laterally while providing resistance to the lateral knee. Pain on this maneuver is suggestive of acute appendicitis. ^7^  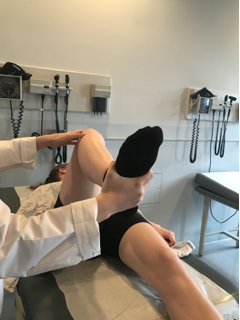 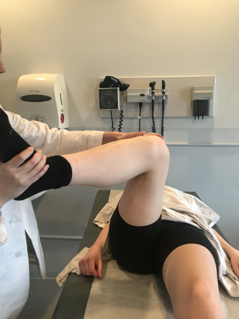  ***Photos: Sandra K. Oza, MD, MA***  ***Discuss the approach to (but do not practice) a pelvic examination:*** Review the steps of the pelvic examination with students that they learned in the ICM-2 course. They were taught the 3 main components of the pelvic exam.   1. Inspection and palpation of the vulva: Assess all structures    1. Mons pubis    2. Labia majora    3. Labia minora    4. Perineum 2. Speculum examination:    1. Technique for insertion       1. Inform patient before beginning, ask patient to bear down       2. Separate labia with index and middle fingers of one hand       3. Choose correct size speculum with other hand       4. Handles are posterior at 45 degrees       5. Insert closed, lubricated speculum through introitus to full length       6. Rotate speculum back 45 degrees until handles in posterior position    2. Visualize and inspect cervix, vagina 3. Bimanual digital examination    1. Labia gently parted with index and middle finger of one hand    2. Lubricated index and middle finger of other hand inserted into vaginal canal    3. Assess vaginal walls, locate cervix    4. Assess cervix, uterus, adnexal structures |
| --- |

HDPE
Case #3: Child with fever

***FACILITATOR NOTES: Ask one student to volunteer to read the clinical vignette for the group. Once the vignette has been read, ask the students to break up into two groups of 4-5 students to complete response to prompts, and then convene a full group discussion of student responses to prompts 1-2 for about 10 minutes. Then, move on to physical exam practice for 10 minutes. Ask students to break up into pairs (or, at most triads) to practice the suggested physical examination maneuvers for this case. While students are practicing, your role is to circulate around to answer questions as well as observe and provide feedback on physical examination technique.***

| **Clinical Vignette**  A 6-year-old girl with cerebral palsy who is non-verbal and wheelchair bound presents with 1 day of fever and fatigue. |
| --- |

| **1. Using the information available to you above, please list some potential diagnoses that could lead to this presentation.**  ***FACILITATOR NOTES:***  ***During the large group discussion, ask each student to provide one possible diagnosis. Do 1-2 rounds time permitting. Following the large group discussion of a differential diagnosis, discuss with students possible frameworks for generating a differential diagnosis?***  *Fever in a patient who is unable to communicate symptoms is usually approached by thinking of infectious and non-infectious causes of fever.*  *Physical Examination is a particularly useful tool in making diagnoses in non-verbal patients. Additionally, please reinforce the importance of gathering history from as many other collateral sources as possible.*  *Infectious etiologies: Otitis media, pharyngitis, bronchitis, pneumonia, skin and soft tissue infection (including skin breakdown in pressure areas), intra-abdominal process, urinary tract infection/pyelonephritis.*  *Inflammatory etiologies: Kawasaki disease.*  *Hematologic Disease: Leukemia/lymphoma* |
| --- |

| **2. Hypothesis-Driven PE: Using the list of diagnoses you identified above for this patient, which physical examination maneuvers would you plan to perform on this patient? Please fill out the table below justifying why you would be doing that maneuver and what you’d be looking for? You do not need to fill in the entire table, and can add rows if needed.**  ***FACILITATOR NOTES: During the large group discussion, ask each student to provide one PE maneuver with justification. Do 1-2 rounds time permitting. A suggested approach to the physical examination for the above case, along with a justification for each of the PE maneuvers, follows below. Encourage students to be as specific as possible with the justification for each of the PE maneuvers they recommend in the evaluation of this patient.***   \| Physical Exam Maneuver \| Justification (what are you looking for?) \| \| --- \| --- \| \| *Vital Signs* \| *BP – low BP might indicate sepsis or other types of shock (anaphylactic)*  *HR- tachycardia could be indicative of systemic inflammatory response syndrome (SIRS).*  *RR and O2 Sat- a high resp rate and/or a low O2 sat would potentially indicate an intrapulmonary process like bronchitis or pneumonia.* \| \| *General Exam* \| *Respiratory pattern/presence of distress:*  *How severe/acute is the presentation?* \| \| *External Eye* \| *Conjunctival injection/conjunctivitis* \| \| *Neck* \| *Is the neck supple or stiff? Check Kernig, Brudzinski sign*  *Lymph node exam- perhaps important, perhaps not – is malignancy a possibility?* \| \| *Ear* \| *Visualize both TMs- look for evidence of Otitis media or externa.* \| \| *Mouth and pharynx* \| *Look for evidence of pharyngitis, tonsillitis and/or dental caries.* \| \| *Cardiac* \| *Auscultation to assess for murmurs – could this be endocarditis?* \| \| *Respiratory* \| *Auscultation, percussion only if abnormality present. – crackles might indicate a pneumonia* \| \| *GI/Abdomen* \| *Auscultation and palpation: looking for any indication of intra-abdominal pathology* \| \| *Skin exam* \| *Look for rashes, including cellulitis or abscesses. Also check area of pressure for skin breakdown and infected ulcers or osteomyelitis.* \| |
| --- | --- | --- | --- | --- | --- | --- | --- | --- | --- | --- | --- | --- | --- | --- | --- | --- | --- | --- | --- | --- | --- | --- |

| **Physical Examination Practice: Please work with a peer in your small group to practice the following physical examination maneuvers relevant to this case:**   1. Examine the outer ear and then the tympanic membrane using an otoscope and speculum. 2. Examine the oropharynx using a tongue depressor. 3. Palpate cervical lymph nodes 4. Perform Kernig and Brudzinki signs 5. Examine the skin for rashes. Discuss with your partner where you might look for skin breakdown (sacral, back of head/ears, heels) Note- students will not check each other’s sacral area.   ***FACILITATOR NOTES: Suggestions for performance of selected PE maneuvers follows below.***  ***Examine the tympanic membrane:*** Students may hold the otoscope either in the dominant hand, or in the same hand as the ear they are about to examine. With the other hand pull the pinna up, out, and back. With the otoscope hand brace the 5th digit on the face of the patient and insert the otoscope while visualizing the canal.^8^  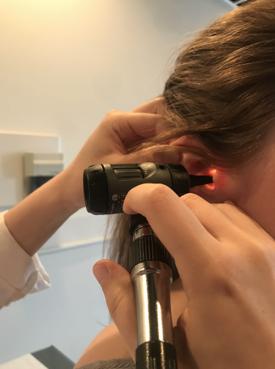  ***Photos: Sandra K. Oza, MD, MA***  ***Examine the oropharynx using a tongue depressor:*** Ask to open their mouth widely while sticking out their tongue. Using a tongue depressor the examiner should press down on the middle third of the tongue, scooping it towards the front teeth. Putting the tongue depressor too far back may stimulate a gag reflex. Be cautious of avoiding compressing the patient’s lower lip with the anterior portion of the tongue depressor. Use a light source to examine the patients oropharynx.^9^  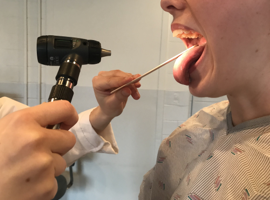  ***Photo: Sandra K. Oza, MD, MA***  ***Palpate cervical lymph nodes:*** The examiner should use the pads of their fingers in a circular motion to palpate the cervical lymph nodes. First palpate the occipital area. Next move to the posterior auricular area, posterior triangle, and along the sternocleidomastoid muscle (first superficially then hooking around the sternocleidomastoid). Finally palpate the anterior triangle region, the submaxillary chain under the jaw, the submental area under the chin, and the anterior auricular area in front of the ear.^10^  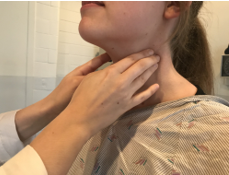 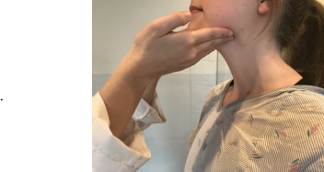  ***Photos: Sandra K. Oza, MD, MA***  ***Perform Kernig’s and Brudzinski’s signs***: For Kernig’s sign the patient should be in a supine position. The examiner should flex the patient’s hip to 90 degrees and attempt to straighten this leg at the knee. Inability to straighten the leg is suggestive of meningitis. For Brudzinski sign the patient should also be supine. The patient’s neck should be passively flexed. A positive sign would occur if neck flexion causes the patients hips and knees to flex. This is suggestive of meningitis.^11^  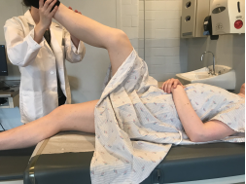 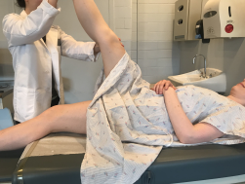  **Checking for Kernig’s Sign**  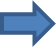 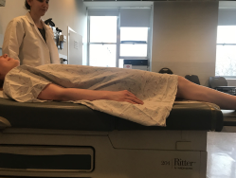 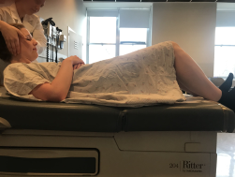  **Positive Brudzinski Sign**  ***Photos: Sandra K. Oza, MD, MA*** |
| --- |

HDPE
Case #4: Irregular Menses

***FACILITATOR NOTES: Ask one student to volunteer to read the clinical vignette for the group. Once the vignette has been read, ask the students to break up into two groups of 4-5 students to complete response to prompts, and then convene a full group discussion of student responses to prompts 1-2 for about 10 minutes. Then, move on to physical exam practice for 10 minutes. Ask students to break up into pairs (or, at most triads) to practice the suggested physical examination maneuvers for this case. While students are practicing, your role is to circulate around to answer questions as well as observe and provide feedback on physical examination technique.***

| **Clinical Vignette:**  A 29-year-old woman with no past medical history presents with several months of irregular menses. Menses started at age 12 and were regular, occurring monthly, and lasting 4 days. 6 months ago the patient experienced heavy menstrual bleeding lasting 7 days. She subsequently did not have menses for 8 weeks, and again had a heavy menses for 7 days. She has only had menses one other time since then and it was light and only lasted 2 days. |
| --- |

| **1. Using the information available to you above, please list some potential diagnoses that could lead to this presentation.**  ***FACILITATOR NOTES:***  ***During the large group discussion, ask each student to provide one possible diagnosis. Do 1-2 rounds time permitting. Following the large group discussion of a differential diagnosis, discuss with students possible frameworks for generating a differential diagnosis?***  *Anatomical and endocrine approach: is there something that would prevent expulsion of blood from the uterus or something that disrupts normal monthly hormonal signaling leading to a lack of normal monthly uterine thickening.*  *Endocrine disorder [Polycystic Ovary Syndrome (PCOS), hyper or hypothyroidism, Cushing Syndrome/Disease,*  *Genetic disorder (Turner Syndrome)*  *Eating disorder (anorexia or bulimia)*  *Pregnancy*  *Virilizing Tumors*  *Anatomical Disorders (congenital uterine malformations, fibroid tumors,*  *Ovarian mass* |
| --- |

| **2. Hypothesis-Driven PE: Using the list of diagnoses you identified above for this patient, which physical examination maneuvers would you plan to perform on this patient? Please fill out the table below justifying why you would be doing that maneuver and what you’d be looking for? You do not need to fill in the entire table, and can add rows if needed.**  ***FACILITATOR NOTES: During the large group discussion, ask each student to provide one PE maneuver with justification. Do 1-2 rounds time permitting. A suggested approach to the physical examination for the above case, along with a justification for each of the PE maneuvers, follows below. Encourage students to be as specific as possible with the justification for each of the PE maneuvers they recommend in the evaluation of this patient.***   \| Physical Exam Maneuver \| Justification (what are you looking for?) \| \| --- \| --- \| \| *Vital Signs* \| *BP – High BP may be associated with hyperthyroidism, adrenal hyperplasia, or PCOS*  *HR- tachycardia could be indicative of hyperthyroidism or hyper adrenalism. Bradycardia could be related to hypothyroidism.*  *Temp: high temp 🡪 hyperthyroidism, low temp 🡪 Hypothyroidism.* \| \| *General Exam* \| *Respiratory pattern/presence of distress:*  *How severe/acute is the presentation?* \| \| *Eye Exam* \| *Look for exophthalmos, lid lag (hyperthyroidism)* \| \| *Oral Exam* \| *Look for signs of bulimia.* \| \| *Neck* \| *Thyroid exam*  *Lymph node exam- perhaps important, perhaps not – is malignancy a possibility?* \| \| *Cardiac* \| *Auscultation. Baseline* \| \| *Respiratory* \| *Auscultation. Baseline* \| \| *GI/Abdomen* \| *Gravid uterus*  *Abdominal masses (intra abdominal malignancy).* \| \| *Neurologic* \| *Cranial nerve II: Visual Fields 🡪 Pituitary compression (homonymous hemianopsia)*  *Cranial nerve III, IV, VI: Extraocular movements*  *Deep tendon reflexes: hyperreflexic (hyperthyroidism), “hung reflexes” (hypothyroidism)* \| |
| --- | --- | --- | --- | --- | --- | --- | --- | --- | --- | --- | --- | --- | --- | --- | --- | --- | --- | --- | --- | --- |

| **Physical Examination Practice: Please work with a peer in your small group to practice the following physical examination maneuvers relevant to this case:**   1. Assess visual fields by confrontation 2. Perform an examination of the thyroid 3. Assess deep tendon reflexes (Facilitator note: This is not a particularly useful examination maneuver for this patient, but is included because student’s often need more practice and feedback on the use of the reflex hammer and assessment of reflexes)   ***FACILITATOR NOTES: Suggestions for performance of selected PE maneuvers follows below.***  ***Assess visual fields by confrontation***: Begin by sitting at eye level across from the patient, approximately 3 feet away. Both patient and examiner should focus on the other’s nose. The patient should cover one eye and the examiner should close their opposite eye. Next, the examiner will hold up both hands, slightly closer to themselves than the patient. The examiner should show one or two fingers in both hands and ask the patient how many fingers they see. The examiner should be able to see their own fingers during this process. This should be done for upper and lower visual fields for both eyes.^12^  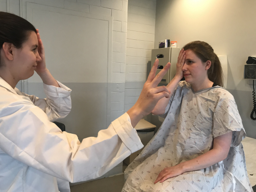 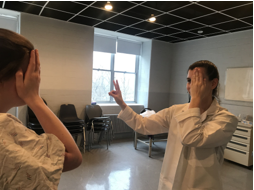  ***Photos: Sandra K. Oza, MD, MA***  ***Perform an examination of the thyroid:***  Anterior Approach: The examiner and patient should sit face to face and the patient should slightly flex their neck towards the side being examined. If examining the right side the examiner should move the larynx to the patient’s left with their left hand. The patient should be asked to swallow while the examiner uses their right hand to palpate for the thyroid on left side of the patient’s neck between the midline and the left sternocleidomastoid muscle below the thyroid cartilage. This should then be repeated on the opposing side.  Posterior Approach: The examiner should stand to the side of the patient, remaining in their peripheral vision. After describing the exam to the patient, the examiner should place their hands around the patient's neck while slightly extended. If examining the right side of the thyroid the examiner should push the trachea to the right with their left hand. The patient should be asked to swallow while the examiners right hand palpates the area between the midline and the right sternocleidomastoid muscle below the thyroid cartilage. This should then be repeated on the opposing side.^13^  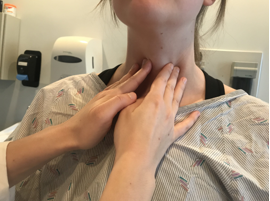 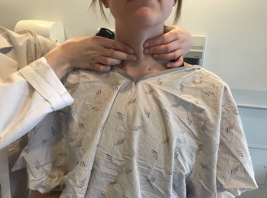  Anterior Approach Posterior Approach  ***Photos: Sandra K. Oza, MD, MA*** |
| --- |

HDPE

Case #5: Dizziness

***FACILITATOR NOTES: Ask one student to volunteer to read the clinical vignette for the group. Once the vignette has been read, ask the students to break up into two groups of 4-5 students to complete response to prompts, and then convene a full group discussion of student responses to prompts 1-2 for about 10 minutes. Then, move on to physical exam practice for 10 minutes. Ask students to break up into pairs (or, at most triads) to practice the suggested physical examination maneuvers for this case. While students are practicing, your role is to circulate around to answer questions as well as observe and provide feedback on physical examination technique.***

| **Clinical vignette**:  78-year-old man with past medical history of hypertension, diabetes mellitus type 2, hyperlipidemia, chronic kidney disease stage 3, coronary artery disease s/p MI 3 years ago, BPH presents to urgent care with complaints of one week of dizziness.  Medications:   - Aspirin - Atorvastatin - Hydrochlorothiazide - Insulin glargine - Insulin lispro - Losartan - Metoprolol - Tamsulosin |
| --- |

| **1. Using the information available to you above, please list some potential diagnoses that could lead to this presentation.**  ***FACILITATOR NOTES:***  ***During the large group discussion, ask each student to provide one possible diagnosis. Do 1-2 rounds time permitting. Following the large group discussion of a differential diagnosis, discuss with students possible frameworks for generating a differential diagnosis?***  *Dizziness is a common presenting complaint to both primary care and emergency department settings. The term dizziness itself can mean different things to different people.*  *There are several approaches one can take in the evaluation of “dizziness.” A careful history can often classify the dizziness into one of four categories as well as several diagnoses that fall into those categories: Vertigo (which can be further divided into peripheral and central vertigo), disequilibrium, pre-syncope and lightheadedness.*   \| *Vertigo: Peripheral*   - *Benign paroxysmal positional vertigo (BPPV)* - *Vestibular neuritis (viral infection of the vestibular nerve)* - *Labyrinthitis (infection of the labyrinthine organs)* - *Meniere’s disease (increased endolymphatic fluid in the inner ear)* - *Cholesteatoma* - *Herpes zoster oticus* - *Otosclerosis*   *Vertigo: Central*   - *Vestibular migraine/migrainous vertigo* - *Cerebellopontine angle tumor* - *CVA/TIA* - *Multiple sclerosis* \| *Disequilibrium:*   - *CVA/TIA* - *Parkinson’s disease* - *Peripheral neuropathy* - *Polypharmacy* \| \| --- \| --- \| \| *Pre-syncope:*   - *Arrhythmias (i.e. SVT)* - *Myocardial infarction* - *Carotid artery stenosis/vertebrobasilar insufficiency* - *Orthostatic hypotension* \| *Lightheadedness:*   - *Hyperventilation* - *Anxiety/panic disorder* - *Hypoglycemia* \| |
| --- | --- | --- | --- | --- |

| **2. Hypothesis-Driven PE: Using the list of diagnoses you identified above for this patient, which physical examination maneuvers would you plan to perform on this patient? Please fill out the table below justifying why you would be doing that maneuver and what you’d be looking for? You do not need to fill in the entire table, and can add rows if needed.**  ***FACILITATOR NOTES: During the large group discussion, ask each student to provide one PE maneuver with justification. Do 1-2 rounds time permitting. A suggested approach to the physical examination for the above case, along with a justification for each of the PE maneuvers, follows below. Encourage students to be as specific as possible with the justification for each of the PE maneuvers they recommend in the evaluation of this patient.***   \| PE Maneuver \| Justification (what are you looking for)? \| \| --- \| --- \| \| *Vital signs* \| *BP: check with patient in supine position and then again in seated and/or standing position at least 1 minute after position change – assess for orthostatic change (SBP -20mmHg or DBP -10mmHg)*  *HR: check with patient in supine position and then again in seated and/or standing position at least 1 minute after position change – assess for orthostatic change (HR +30bpm)* \| \| *HEENT* \| *Oropharynx: mucous membranes moist/dry (hypovolemia, orthostatic hypotension)* \| \| *Cardiac* \| *Auscultation: cardiac rhythm (arrhythmias)*  *Auscultation of carotid artery for bruits* \| \| *Neurologic exam: CN* \| *III, IV, VI: Assess for nystagmus on primary or lateral gaze*  *VIII: assess gross hearing (Meniere’s disease), head impulse test (see below)* \| \| *Neurologic exam: Motor* \| *Muscle tone: increased in Parkinson’s, upper motor neuron lesions (i.e. CVA)*  *Muscle strength: evaluate for weakness (CVA)* \| \| *Neurologic exam: Sensory* \| *Assessing for sensory deficits – (CVA, peripheral neuropathy)* \| \| *Neurologic exam: DTR* \| *Assessing for hyperreflexia – CVA* \| \| *Neurologic exam: Cerebellum* \| *Romberg: swaying/falling to one side suggests ipsilateral vestibular dysfunction*  *Gait: pace and arm swing (slower pace and reduced arm swing in Parkinson’s; gait in cerebellar ataxia is slow, wide based and irregular)* \| \| *Dix-Hallpike* \| *Assessment for nystagmus – sensitivity for BPPV is 50-88%* \| \| *Head impulse test* \| *Assessment for catch up saccade – CN VIII problem (i.e. vestibular neuritis)* \| |
| --- | --- | --- | --- | --- | --- | --- | --- | --- | --- | --- | --- | --- | --- | --- | --- | --- | --- | --- | --- | --- | --- | --- |

| **Physical Examination Practice: Please work with a peer in your small group to practice the following physical examination maneuvers relevant to this case:**   1. Measure blood pressure and heart rate; discuss the approach to measuring orthostatic vital signs. (In the interest of time, have all students practice blood pressure in one position, but please just have one group demonstrate proper technique for orthostatic vital signs) 2. Perform the following components of the neurologic examination:    1. Assess cranial nerves    2. Perform a sensory examination of the upper and lower extremities    3. Perform a motor examination of the upper and lower extremities    4. Perform a Romberg maneuver    5. Assess cerebellar function with finger-to-nose and heel-to-shin testing 3. Perform a Dix-Hallpike maneuver   ***FACILITATOR NOTES: Suggestions for performance of selected PE maneuvers follows below.***  ***Measure blood pressure and heart rate; discuss the approach to measuring orthostatic vital signs.*** Have the patient lie down for at least 5 minutes and then measure the baseline blood pressure and pulse. Then have the patient stand up and repeat these measurements immediately.  *Orthostatic hypotension* is defined as a drop in systolic blood pressure of 20 mm Hg or more or a drop in diastolic blood pressure of 10 mm Hg or more when a patient stands. The patient also experiences symptoms such as dizziness, lightheadedness, and syncope. There is usually an increase in heart rate.^14^  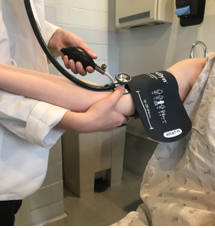  ***Photo: Sandra K. Oza, MD, MA***  ***Perform a Romberg maneuver:*** Have the patient stand with feet together. Stand close to the patient during this maneuver. Ask them to close their eyes. The test is positive if the patient must move their feet to balance and suggests a problem with the dorsal columns.^15^  ***Assess coordination with finger-to-nose and heel-to-shin testing***:  Finger to Nose: Stand in front of the patient and ask them to touch their nose and then the examiner’s finger, repeating as you move your finger to different points. ^15^  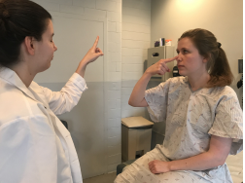 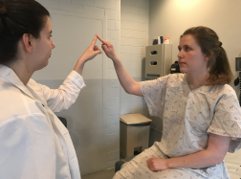  ***Photos: Sandra K. Oza, MD, MA***  Heel to shin: Ask the patient to lie supine and slide the heel of one foot down the opposite leg from knee to ankle. A normal result would be smooth motion of the heel along the shin without removing it from the shin. ^15^  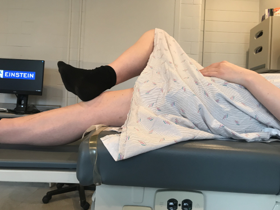 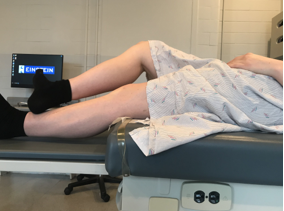  ***Photos: Sandra K. Oza, MD, MA***  ***Perform a Dix-Hallpike maneuver:***  Setup: Begin with the patient seated at the edge of the examining table such that when they lie backwards their head will be slightly off the table behind them. Turn the patients head to 45 degrees from their sagittal plane and to the side being tested. Inform the patient that this may reproduce sensations of dizziness or nausea. Advise the patient to keep their eyes open throughout the maneuver.  Maneuver: Quickly move the patient into a supine position with their head hanging approximately 20 degrees off the table and neck extended slightly so that the chin is up, but maintain their head at the original 45 degree angle to their sagittal plane. Observe the patient in this position for at least 30 seconds, with eyes open. Watch eyes for the direction, duration, and latency of nystagmus. Note if there is any reproduction of symptoms.  Repeat the maneuver with the head turned toward the other side.^16^    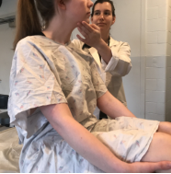 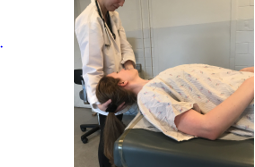 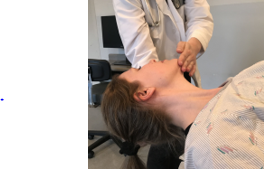  ***Photos: Sandra K. Oza, MD, MA*** |
| --- |

HDPE
Wrap-up

***FACILITATOR NOTES: Convene a brief large-group discussion with the time remaining. Pose the following discussion questions to students.***

*How will students use this activity moving forward?*

*When might it be appropriate to just do a complete physical exam and not worry about the approach towards a focused physical exam?*

*What was most challenging about this activity?*

*What physical exam maneuvers do you still need more practice with?*

***THANK YOU FOR FACILITATING! We would love your feedback on this session.***

References:

1. Swartz MH. The Heart. In: Swartz MH, ed. *Textbook of physical diagnosis: history and examination*. 7^th^ Ed. Philadelphia, PA: Elsevier Saunders; 2014:370-72
2. Swartz MH. The Heart. In: Swartz MH, ed. *Textbook of physical diagnosis: history and examination*. 7^th^ Ed. Philadelphia, PA: Elsevier Saunders; 2014:376-77
3. Swartz MH. The Heart. In: Swartz MH, ed. *Textbook of physical diagnosis: history and examination*. 7^th^ Ed. Philadelphia, PA: Elsevier Saunders; 2014:373-374
4. Swartz MH. The Skin. In: Swartz MH, ed. *Textbook of physical diagnosis: history and examination*. 7^th^ Ed. Philadelphia, PA: Elsevier Saunders; 2014:90
5. Swartz MH. The Abdomen. In: Swartz MH, ed. *Textbook of physical diagnosis: history and examination*. 7^th^ Ed. Philadelphia, PA: Elsevier Saunders; 2014:452-54
6. Swartz MH. The Abdomen. In: Swartz MH, ed. *Textbook of physical diagnosis: history and examination*. 7^th^ Ed. Philadelphia, PA: Elsevier Saunders; 2014:456-57
7. Hardin DM. Acute Appendicitis: Review and Update. *Am Fam Physician*. 1999 Nov 1;60(7):2027-2034.
8. Swartz MH. The Ear and Nose. In: Swartz MH, ed. *Textbook of physical diagnosis: history and examination*. 7^th^ Ed. Philadelphia, PA: Elsevier Saunders; 2014:266
9. Swartz MH. The Oral Cavity and Pharynx. In: Swartz MH, ed. *Textbook of physical diagnosis: history and examination*. 7^th^ Ed. Philadelphia, PA: Elsevier Saunders; 2014:302
10. Swartz MH. The Head and Neck. In: Swartz MH, ed. *Textbook of physical diagnosis: history and examination*. 7^th^ Ed. Philadelphia, PA: Elsevier Saunders; 2014:149’
11. Bickley LS, Szilagyi PG, Hoffman RM. The Nervous System. In: Bickley LS, Szilagyi PG, Hoffman RM, ed. *Bates’ guide to physical examination and history taking*. 12th Ed. Philadelphia, PA: Wolters Kluwer; 2017:765
12. Swartz MH. The Eye. In: Swartz MH, ed. *Textbook of physical diagnosis: history and examination*. 7^th^ Ed. Philadelphia, PA: Elsevier Saunders; 2014:174
13. Swartz MH. The Skin. In: Swartz MH, ed. *Textbook of physical diagnosis: history and examination*. 7^th^ Ed. Philadelphia, PA: Elsevier Saunders; 2014:153-4
14. Swartz MH. The Heart. In: Swartz MH, ed. *Textbook of Physical Diagnosis: History and Examination*. 7^th^ Ed. Philadelphia, PA: Elsevier Saunders; 2014:366-7
15. Swartz MH. The Nervous System. In: Swartz MH, ed. *Textbook of physical diagnosis: history and examination*. 7^th^ Ed. Philadelphia, PA: Elsevier Saunders; 2014:628-9
16. Munchi H, Sirmans SM, James E. Dizziness: Approach to Evaluation and Management. *Am Fam Physician*. 2017 Feb 1;95(3):154-162.
