# Supplementary material for: Implementation of a Hypothesis-Driven Physical Exam Session in a Transition to Clerkship Program
Source: MedEdPORTAL. 2020 Nov 24;16:11043. doi: 10.15766/mep_2374-8265.11043 (PMC7703480; doi:10.15766/mep_2374-8265.11043)
Supplement: Supplementary file 1 — Student Worksheet.docxFacilitator Guide.docxPostsession Student Survey.docxPostsession Facilitator Survey.docxFour-Month Follow-Up Student Survey.docx [file mep_2374-8265.11043-s001.zip › C. Postsession Student Survey.docx]

**Appendix C** – Post Session Student Survey

| 1. After the Session I have achieved the following learning objectives: | | | | |
| --- | --- | --- | --- | --- |
|  | Not at All | Slightly | Moderately | Completely |
| Create a quick, yet broad differential diagnosis for a patient vignette |  |  |  |  |
| Determine which physical exam maneuvers are most applicable for patients presenting with common symptoms using your differential diagnosis and clinical reasoning. |  |  |  |  |
| Justify why a physical exam maneuver should be included or excluded when seeing a patient for a focused problem |  |  |  |  |
| Demonstrate the ability to perform focused physical examination maneuvers on peers. |  |  |  |  |
| Interpret physical examination findings and apply those findings to refine a differential diagnosis for a patient with a common presenting symptom |  |  |  |  |

| 2. Please indicate your level of agreement with the following statements: | | | | |  |
| --- | --- | --- | --- | --- | --- |
|  | N/A | Strongly Disagree | Disagree | Agree | Strongly Agree |
| The session was well organized. |  |  |  |  |  |
| The session allowed me to develop my ability to generate a quick differential diagnosis and receive feedback on it. |  |  |  |  |  |
| This session allowed me to think critically about why I might conduct a certain physical examination maneuver or not based on the patient’s chief complaint and the other active and past problems. |  |  |  |  |  |
| This session allowed me to practice physical examination maneuvers and receive feedback on my technique. |  |  |  |  |  |
| The workshop activities allowed me to reinforce knowledge I already had learned. |  |  |  |  |  |
| The workshop activities allowed me to build new knowledge and/or make new connections in my learning. |  |  |  |  |  |
| I believe this session will help me as a third year clerk. |  |  |  |  |  |
| I am confident about my physical exam skills as I enter the clerkship year. |  |  |  |  |  |
| I am confident I can determine which physical examination maneuvers are most important to perform on a patient with a particular chief complaint. |  |  |  |  |  |

| 3. Please rate your confidence in the ability to perform the following skills **BEFORE** this session | | | | |  |
| --- | --- | --- | --- | --- | --- |
|  | N/A | Not at All | A little | Moderately | Very |
| Perform a problem focused examination |  |  |  |  |  |
| Use a differential diagnosis in real-time to determine what physical examination maneuvers might be high yield to perform in a patient with a particular chief complaint |  |  |  |  |  |
| Have an organized approach to the physical examination that should be conducted in a patient presenting with shortness of breath |  |  |  |  |  |
| Have an organized approach to the physical examination that should be conducted in a patient presenting with fever |  |  |  |  |  |
| Have an organized approach to the physical examination that should be conducted in a patient presenting with irregular menses |  |  |  |  |  |
| Have an organized approach to the physical examination that should be conducted in a patient presenting with abdominal pain |  |  |  |  |  |
| Have an organized approach to the physical examination that should be conducted in a patient presenting with dizziness |  |  |  |  |  |
| Have an organized approach to the physical examination that should be conducted in a patient presenting with headache |  |  |  |  |  |
| Have an organized approach to the physical examination that should be conducted in a patient presenting with fatigue |  |  |  |  |  |

| 4. Please rate your confidence in the ability to perform the following skills **AFTER** this session | | | | |  |
| --- | --- | --- | --- | --- | --- |
|  | N/A | Not at All | A little | Moderately | Very |
| Perform a problem focused examination |  |  |  |  |  |
| Use a differential diagnosis in real-time to determine what physical examination maneuvers might be high yield to perform in a patient with a particular chief complaint |  |  |  |  |  |
| Have an organized approach to the physical examination that should be conducted in a patient presenting with shortness of breath |  |  |  |  |  |
| Have an organized approach to the physical examination that should be conducted in a patient presenting with fever |  |  |  |  |  |
| Have an organized approach to the physical examination that should be conducted in a patient presenting with irregular menses |  |  |  |  |  |
| Have an organized approach to the physical examination that should be conducted in a patient presenting with abdominal pain |  |  |  |  |  |
| Have an organized approach to the physical examination that should be conducted in a patient presenting with dizziness |  |  |  |  |  |
| Have an organized approach to the physical examination that should be conducted in a patient presenting with headache |  |  |  |  |  |
| Have an organized approach to the physical examination that should be conducted in a patient presenting with fatigue |  |  |  |  |  |

*Free Response Questions:*

1. Please indicate one thing you learned during this session.
2. Please indicate a concept addressed in this session that remains unclear or that you would like to learn more about.
3. What other chief complaints would you have wanted to see covered in this session?
4. How can you forsee using this session in your third year clerkships?
5. Which physical examination maneuvers do you feel most comfortable performing?
6. Which physical examination maneuvers do you feel least comfortable performing? Which do you feel least comfortable interpreting?
7. How does considering a differential diagnosis before performing a physical examination impact how you view the role of the physical examination in clinical care?
8. Please comment on the strengths of the session.
9. Please comment on what could make this session better.
