# Supplementary material for: Implementation of a Hypothesis-Driven Physical Exam Session in a Transition to Clerkship Program
Source: MedEdPORTAL. 2020 Nov 24;16:11043. doi: 10.15766/mep_2374-8265.11043 (PMC7703480; doi:10.15766/mep_2374-8265.11043)
Supplement: Supplementary file 1 — Student Worksheet.docxFacilitator Guide.docxPostsession Student Survey.docxPostsession Facilitator Survey.docxFour-Month Follow-Up Student Survey.docx [file mep_2374-8265.11043-s001.zip › D. Postsession Facilitator Survey.docx]

**Appendix D** – Post Session Facilitator Survey

| 1. Please indicate how well you feel the students achieved the following learning objectives for the session: | | | | |
| --- | --- | --- | --- | --- |
|  | Not at All | Slightly | Moderately | Completely |
| Create a quick, yet broad differential diagnosis for a patient vignette |  |  |  |  |
| Determine which physical exam maneuvers are most applicable for patients presenting with common symptoms using your differential diagnosis and clinical reasoning. |  |  |  |  |
| Justify why a physical exam maneuver should be included or excluded when seeing a patient for a focused problem |  |  |  |  |
| Demonstrate the ability to perform focused physical examination maneuvers on peers. |  |  |  |  |
| Interpret physical examination findings and apply those findings to refine a differential diagnosis for a patient with a common presenting symptom |  |  |  |  |

| 2. Please rate the value of: | | | | |
| --- | --- | --- | --- | --- |
|  | Not Valuable at all | Somewhat Valuable | Moderately Valuable | Extremely Valuable |
| The facilitator guide in helping you run this small group session |  |  |  |  |
| The just in time training in helping you run this small group session |  |  |  |  |

|  | | | | |
| --- | --- | --- | --- | --- |
|  | Not enough time and we finished late | Not enough time but we managed to finish on time | Just the right amount of time/we finished on time | Ample time to complete the activity and we got out early |
| 3. Please rate the timing to accomplish learning activities in this session: |  |  |  |  |

|  | | | | |  |  |  |
| --- | --- | --- | --- | --- | --- | --- | --- |
|  | Not Valuable at all | Somewhat Valuable | Moderately Valuable | Extremely Valuable |  |  |  |
| 4. Please rate your satisfaction with student group size and your ability to perform direct observation. |  |  |  |  |  |  |  |

|  | Extremely Dissatisfied | Very Dissatisfied | Slightly Dissatisfied | Neither Satisfied nor dissatisfied | Slightly Satisfied | Very Satisfied | Extremely Satisfied |
| --- | --- | --- | --- | --- | --- | --- | --- |
| 5. How satisfied or dissatisfied were you with teaching in this session? |  |  |  |  |  |  |  |

*Free Response Questions:*

1. What part of this session was most challenging for your student group?
2. Which physical examination maneuvers did students seem to have trouble with?
3. How important was the physical examination practice (hands on time with peers) in this session? Do you think it would have been better without the PE practice and just focusing on clinical reasoning?
4. What is your perception of your student group’s clinical reasoning skills?
5. Please comment on the strengths of this session.
6. What would have made this session better?
7. What other chief complaints would you have wanted to see covered in this session?
8. Please leave any other comments you have about the hypothesis driven physical examination session.
