# Supplementary material for: Implementation of a Hypothesis-Driven Physical Exam Session in a Transition to Clerkship Program
Source: MedEdPORTAL. 2020 Nov 24;16:11043. doi: 10.15766/mep_2374-8265.11043 (PMC7703480; doi:10.15766/mep_2374-8265.11043)
Supplement: Supplementary file 1 — Student Worksheet.docxFacilitator Guide.docxPostsession Student Survey.docxPostsession Facilitator Survey.docxFour-Month Follow-Up Student Survey.docx [file mep_2374-8265.11043-s001.zip › E. Four-Month Follow-Up Student Survey.docx]

|  | N/A | Poor | Satisfactory | Good | Exceptional |
| --- | --- | --- | --- | --- | --- |
| 1.Rate the usefulness of the hypothesis driven physical examination session. |  |  |  |  |  |

**Appendix E** – 4-month Follow-up Student Survey

| 2. Please rate your confidence in the ability to perform the following skills: | | | | |  |
| --- | --- | --- | --- | --- | --- |
|  | N/A | Not at All | A little | Moderately | Very |
| Perform a problem focused examination |  |  |  |  |  |
| Use a differential diagnosis in real-time to determine what physical examination maneuvers might be high yield to perform in a patient with a particular chief complaint |  |  |  |  |  |
| Have an organized approach to the physical examination that should be conducted in a patient presenting with shortness of breath |  |  |  |  |  |
| Have an organized approach to the physical examination that should be conducted in a patient presenting with fever |  |  |  |  |  |
| Have an organized approach to the physical examination that should be conducted in a patient presenting with irregular menses |  |  |  |  |  |
| Have an organized approach to the physical examination that should be conducted in a patient presenting with abdominal pain |  |  |  |  |  |
| Have an organized approach to the physical examination that should be conducted in a patient presenting with dizziness |  |  |  |  |  |
| Have an organized approach to the physical examination that should be conducted in a patient presenting with headache |  |  |  |  |  |
| Have an organized approach to the physical examination that should be conducted in a patient presenting with fatigue |  |  |  |  |  |
